# Chromatin Remodeling in Patient‐Derived Colorectal Cancer Models

**DOI:** 10.1002/advs.202303379

**Published:** 2024-02-21

**Authors:** Kun Xiang, Ergang Wang, John Mantyh, Gabrielle Rupprecht, Marcos Negrete, Golshid Sanati, Carolyn Hsu, Peggy Randon, Anders Dohlman, Kai Kretzschmar, Shree Bose, Nicholas Giroux, Shengli Ding, Lihua Wang, Jorge Prado Balcazar, Qiang Huang, Pasupathi Sundaramoorthy, Rui Xi, Shannon Jones McCall, Zhaohui Wang, Chongming Jiang, Yubin Kang, Scott Kopetz, Gregory E. Crawford, Steven M. Lipkin, Xiao‐Fan Wang, Hans Clevers, David Hsu, Xiling Shen

**Affiliations:** ^1^ Department of Biomedical Engineering Pratt School of Engineering Duke University Durham NC 27708 USA; ^2^ Department of Medicine, School of Medicine Duke University Durham NC 27710 USA; ^3^ Laboratory of Signal Transduction National Institute of Environmental Health Sciences Research Triangle Park Durham NC 27709 USA; ^4^ Oncode Institute Hubrecht Institute Royal Netherlands Academy of Arts and Sciences (KNAW) and University Medical Center (UMC) Utrecht Uppsalalaan 8 Utrecht CT 3584 The Netherlands; ^5^ Mildred Scheel Early Career Centre (MSNZ) for Cancer Research Würzburg University Hospital Würzburg 97080 Würzburg Germany; ^6^ Terasaki Institute Los Angeles CA 90024 USA; ^7^ Department of Pathology School of Medicine Duke University Durham NC 27710 USA; ^8^ Department of Gastrointestinal (GI) Medical Oncology Division of Cancer Medicine The University of Texas MD Anderson Cancer Center Houston TX 77030 USA; ^9^ Department of Pediatrics Division of Medical Genetics, School of Medicine Duke University Durham NC 27710 USA; ^10^ Department of Medicine and Program in Mendelian Genetics Weill Cornell Medicine New York NY 10021 USA; ^11^ Department of Pharmacology and Cancer Biology Duke University Medical Center Durham NC 27710 USA; ^12^ Present address: Roche Pharmaceutical Research and Early Development Basel Switzerland

**Keywords:** ATAC‐seq, Colorectal Cancer (CRC), Patient‐Derived Models of Cancer (PDMC), Patient‐Derived Organoids (PDO), Patient‐Derived Xenografts (PDX)

## Abstract

Patient‐Derived Organoids (PDO) and Xenografts (PDX) are the current gold standards for patient‐derived models of cancer (PDMC). Nevertheless, how patient tumor cells evolve in these models and the impact on drug response remains unclear. Herein, the transcriptomic and chromatin accessibility landscapes of matched colorectal cancer (CRC) PDO, PDX, PDO‐derived PDX (PDOX), and original patient tumors (PT) are compared. Two major remodeling axes are discovered. The first axis delineates PDMC from PT, and the second axis distinguishes PDX and PDO. PDOX are more similar to PDX than PDO, indicating the growth environment is a driving force for chromatin adaptation. Transcription factors (TF) that differentially bind to open chromatins between matched PDO and PDOX are identified. Among them, KLF14 and EGR2 footprints are enriched in PDOX relative to matched PDO, and silencing of KLF14 or EGR2 promoted tumor growth. Furthermore, EPHA4, a shared downstream target gene of KLF14 and EGR2, altered tumor sensitivity to MEK inhibitor treatment. Altogether, patient‐derived CRC cells undergo both common and distinct chromatin remodeling in PDO and PDX/PDOX, driven largely by their respective microenvironments, which results in differences in growth and drug sensitivity and needs to be taken into consideration when interpreting their ability to predict clinical outcome.

## Graphical abstract

Patient‐derived CRC cells undergo two‐axes chromatin remodeling in PDO and PDX/PDOX, largely driven by their respective microenvironments. Transcription factors and genes affected by this remodeling significantly impact tumor growth and drug sensitivity. Therefore, the chromatin adaptation of different PDMC may interfere with their ability to predict therapeutic outcomes.

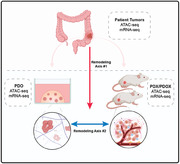

## 1 Introduction

PDMC – such as PDX and PDO – are emerging as powerful avatars of patient tumors for pre‐clinical therapeutic development.^[^ ^1^, ^2^, ^3^, ^4^ ^]^ Compared to cell lines and genetically engineered mouse models, PDX and PDO tend to capture more clinical diversity in terms of disease stage and genetic background.^[^ ^5^ ^]^ PDX and PDO recapitulate drug sensitivity associated with genetic mutations^[^ ^6^, ^7^ ^]^ and correlate with clinical response to chemotherapy.^[^ ^8^, ^9^ ^]^ In therapeutic development, PDX and PDO also help researchers address the high failure rates for new cancer drugs in clinical trials.^[^ ^10^, ^11^ ^]^ However, it remains unclear whether tumor cells undergo changes in PDMC compared to the tumor they were derived from and how the distinct microenvironments of these models may impact patient tumor cell growth and drug sensitivity.

CRC is the 3^rd^ most common cause of cancer‐related death worldwide with ≈150000 new cases diagnosed in the United States annually.^[^ ^12^, ^13^ ^]^ PDX and PDO have been heavily used as pre‐clinical models for CRC research and drug development.^[^ ^14^, ^15^, ^16^, ^17^ ^]^ Recent high‐profile studies from multiple groups consistently showed that PDO can predict CRC patient response to chemotherapy and chemoradiation.^[^ ^18^, ^19^, ^20^ ^]^ However, although molecular signatures of the original tumors are largely recapitulated in those models, CRC cells have been shown to lose their dependency on niche factors as they are passaged,^[^ ^21^ ^]^ indicating their gradual adaptation to the new environment. Therefore, it is important to understand how the evolution of patient CRC cells in PDX and PDO respectively compares to that of the original patient tumor, which can inform which therapeutic axes in PDMC are transferable to clinical outcomes.

In this National Cancer Institute Patient‐Derived Model Consortium‐sponsored study, we compared the chromatin accessibility landscapes between matched sets of PT, PDO, PDX, and PDOX. All three models exert chromatin alterations when compared to PT cells, representing a PT‐PDMC epigenetic axis. Chromatin alterations in CRC cells are more similar between PDOX and PDX than PDO, indicating that the growth environment of the model exerts strong influence on chromatin adaptation in tumor cells. Distinct chromatin alterations and differential TF binding between PDO and PDOX help further delineate the in vitro versus in vivo evolution of CRC, which results in differences in tumor growth and drug sensitivity.

## 2 Results

**Figure 1:**
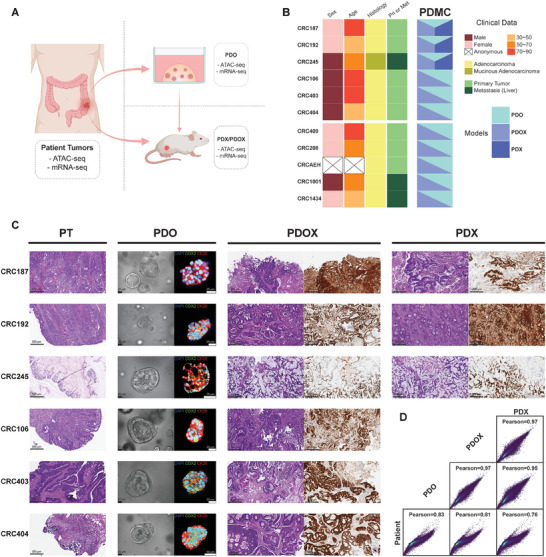
The establishment of matched PT‐PDMC sets. A) The overall scheme of the CRC specimens and PDMC processing. The same surgically removed tumor specimens were used to derive both PDO and PDX. PDO were subcutaneously injected into mice to obtain PDOX. All available models, including the original patient tumors, were processed for ATAC‐seq and mRNA‐seq. B) The summary of model establishment and clinical data for all patients. The bottom five patients were for the additional PDO‐PDOX validation sets. C) Histology of PT and PDMC, showing the sets of CRC187, CRC192, CRC245, CRC106, CRC403, and CRC404. PT panel: H&E‐staining of PT at 10X magnification showing the tumor region. Scale bars, 300 µm; PDO panel: The bright‐field images (left) of PDO. Scale bars, 25 µm. The confocal images (right) of PDO immunolabeled for CDX2 (green), CK20 (red), and DAPI (blue); PDX panel and PDOX panel: H&E‐staining (left) and protein immunostaining for CDX2 (right) of PDX/PDOX. Scale bars, 300 µm. D) Pairwise ATAC‐seq correlation between patient samples and models based on normalized read counts of detected peaks. The Pearson correlation coefficient is presented for each comparison.

For this study, we derived matched PDO, PDX, and PDOX from CRC specimens and performed Assay for Transposase‐Accessible Chromatin using sequencing (ATAC‐seq) and RNA‐seq (**Figure** **1**A). The surgically resected primary and metastatic CRC specimens from consented patients were collected at the Duke BioRepository & Precision Pathology Center (BRPC) (Figure 1B; and Table S1, Supporting Information) and were used to generate corresponding PDO and subcutaneous PDX. We also developed PDOX from the PDO (Figure 1A,C; Figure S1A–E, Supporting Information). The matched sample sets were established from eleven patients across different CRC sub‐types and genomic landscapes (Figure 1B, Table S2, Supporting Information). We performed whole exome sequencing (WES) on PDMC models and found the models represented most mutations of patient tumors (Figure S2A–C, Supporting Information). This is concordant with previous findings that both PDO and PDX preserve most of the genetic landscape of their original tumors.^[^ ^1^, ^22^, ^23^, ^24^ ^]^

We next profiled the open‐chromatin landscapes of the matched complete PT‐PDMC sets using ATAC‐seq^[^ ^25^, ^26^ ^]^ supplemented by messenger RNA sequencing (mRNA‐seq). We obtained high‐quality ATAC‐seq libraries with high consistency across replicates, as reflected by the QC metrics, including overall sequencing depth, mitochondria fractions, and peak numbers (Table S3, Supporting Information). Mouse components from the xenografts were removed via the mouse‐cell‐depletion kit prior to the library preparations so that the average mapping rates to human genome hg19 were >90%.

**Figure 2:**
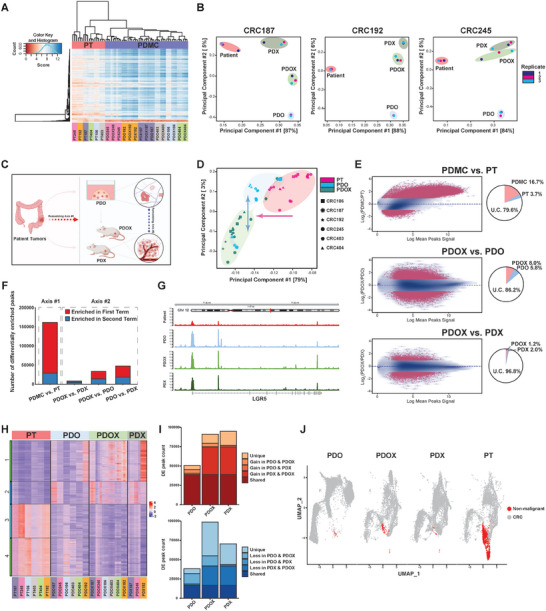
ATAC‐seq analysis suggests a two‐axes remodeling in CRC cells of PDMC. A) Heatmap of unbiased hierarchical clustering of PT and PDMC based on 1000 chromatin accessible regions with the highest variance. Patient tumor and PDMC are separated. All replicates are clustered with each other, and the samples from the same patient set are indicated/labeled with a unique color. B) Principal component analysis of individual PT‐PDMC sets (CRC187, CRC192, and CRC245) from ATAC‐seq data based on the DiffBind score. Patient samples are circled in red, PDO in blue, PDX in green, and PDOX in light green. Each type of model has three replicates. C) The illustration of the two‐axes remodeling. D) Principal component analysis of the pooled PT‐PDO‐PDOX sets from ATAC‐seq data based on the DiffBind score. Patient samples are magenta, PDO are blue, and PDOX are green. Different shapes of dots denote patient IDs. E) Differential analysis of ATAC‐seq peaks in the comparisons of PDMC versus PT, PDOX versus PDO, and PDOX versus PDX based on the consensus peak set derived from each condition. Differentially enriched (DE) peaks are labeled in red, |log2(FC)|>1 and *p* < 0.05. Pie charts show the percentages of enriched or unchanged (U.C.) peaks. F) The number of differentially enriched peaks in different model comparisons. The number of peaks enriched in the first or second term of the comparison is denoted (Table S4, Supporting Information). The remodeling axis #1 includes PDMC versus PT. The remodeling axis #2 includes PDOX versus PDX, PDOX versus PDO, and PDO versus PDX. G) ATAC‐seq signal track showing LGR5 locus in different models. The exon locations are indicated in the gene map. H) Heatmap of all ATAC differentially enriched peaks gained or lost between patient samples, PDO, PDOX, and PDX based on the consensus peak set. Each column represents a replicate of ATAC sequencing of PT or models. I) The number of ATAC‐seq peaks that are significantly gained (top) or lost (bottom) in PDMC versus PT samples, shared, or unique to models. J) Anchored single cell multiome analysis for PDMC and PT, with visualizations of CRC and non‐malignant components.

The global chromatin accessibility landscapes revealed by ATAC‐seq were largely congruent across PT and PDMC (PDO, PDX, and PDOX), suggesting that CRC cells mostly retained their identity in PDMC (Figure 1D; Figures S3 and S4A, Supporting Information). Nevertheless, certain chromatin accessibility alterations could be detected by differential expression analysis based on consensus peaks from PDMC and PT, according to Diffbind,^[^ ^27^ ^]^ resulting in separate PT and PDMC clusters in the hierarchical heatmap (**Figure** **2**A).

Principal component analysis (PCA) of the chromatin accessibility showed clear separations between PT, PDO, and PDX/PDOX (Figure 2B; Figure S5A, Supporting Information). In all cases, the first principal component (PC) axis distinguished PT from all PDMC, and the second PC axis distinguished in vivo models (PDX/PDOX) from the in vitro model (PDO) (Figure 2C). PDOX were more similar to PDX than PDO along the second axis. The pooled PT‐PDMC sets revealed that the two epigenetic axes were still largely preserved despite patient‐to‐patient variation (Figure 2D), and this was also reflected in transcriptomic PCA from mRNA‐seq data (Figure S5B, Supporting Information). PDOX and PDO were also highly correlated versus PT according to both ATAC‐seq and RNA‐seq (Figure S5C,D, Supporting Information), in concordance with the first PCA axis.

Although the majority of consensus peaks remained unchanged (79.6%), paired differential analysis identified alterations in chromatin accessibility between PT and PDMC (Figure 2E,F; Figure S6A,B, Supporting Information), such as at loci in chromosomes 7, 8, 13, and X (Figure S4B, Supporting Information). For instance, there were considerable alterations between PDMC and PT at the locus associated with LGR5, a stem cell and proliferation marker for colon stem cells and CRC^[^ ^28^, ^29^, ^30^, ^31^ ^]^ (Figure 2G). Compared to PT versus PDMC along the first axis, fewer differentially enriched peaks were detected for PDOX versus PDO and PDOX versus PDX along the second axis (Figure 2E,F; Figure S6A,B, Supporting Information). The differentially enriched peaks were the fewest between PDX and PDOX (Figure 2F), in concordance with the PCA analyses (Figure 2B). PDO, PDOX, and PDX had similar overall chromatin accessibilities at the chromosome level, but there were more differences for PDOX versus PDO than PDOX versus PDX.

We performed differential chromatin accessibility analysis on the consensus ATAC‐seq peak set across the matched sets of PT, PDO, PDOX, and PDX. Unsupervised hierarchical clustering of all differentially accessible peaks identified gain/loss clusters that are shared or unique among PDMC (Figure 2H,I). KEGG pathway and Gene Ontology analyses based on gain/loss peaks further suggested pathways that may be commonly or uniquely altered among PDMC (Figure S7A–C, Supporting Information). The pathway analysis on the shared loss peaks suggests PT likely contains more stromal cells than PDMC. We performed 10X Single Cell Multiome (RNA+ATAC) sequencing on the CRC187 set of PT‐PDO‐PDOX‐PDX (Figure S8A, Supporting Information). The human stromal component is higher in PT than in PDMC (Figure 2J; Figure S8B, Supporting Information), which may have contributed to the difference between PT and PDMC. Additionally, we found that the cell clustering and RNA velocity patterns are more similar between PDOX and PDX (Figure S8A,C, Supporting Information), further supporting our finding from the bulk analysis that PDOX is closer to PDX than PDO.

To explore whether the remodeling processes are conserved across different CRC genomic sub‐types, we generated five additional sets of matched PDO‐PDOX (Figure 1B; Figures S1E and S9A–C, Supporting Information) and divided the total 11 PDMC sets into two groups based on their KRAS mutation status (Table S2, Supporting Information). Both wild‐type and mutant KRAS groups showed separate clustering between patient tumor and PDMC based on ATAC‐seq (Figure S10A,B, Supporting Information). PCA (Figure S10C,D, Supporting Information) revealed that PDMC from both groups underwent remodeling along the two axes (PDMC vs patient tumor, and in vivo vs in vitro). These data suggested that the remodeling processes are conserved across sub‐types. Furthermore, differential pathway analysis of PDOX versus PDO demonstrated both shared and distinct PDOX‐enriched pathways between wild‐type and mutant KRAS groups (Figure S10E,F, Supporting Information), suggesting that both the environment and genetic diversity influence the remodeling trajectories.

The findings that PDOX were more similar to PDX than PDO (Figure 2F,H,I) further imply that chromatin alterations were influenced by the tumor environment. KEGG pathways analysis (Figure S11, Supporting Information) suggested pathways involving cell‐cell communication, interactions with the extracellular matrix, and signaling transduction (BRAF, MAPK, EPH‐Ephrin) were altered between PDO and PDOX. Cancer‐associated fibroblasts (CAF) in the tumor microenvironment are known to interact with cancer cells by producing cytokines/chemokines and remodeling the extracellular matrix.^[^ ^32^ ^]^ ATAC‐seq indicates that PDO cocultured with CAF isolated from PDOX (PDO_CAF) are closer to PDOX than the original PDO in terms of the chromatin accessibility landscape (Figure S12A–C, Supporting Information). Pathways, such as the ECM‐receptor interaction pathway and focal adhesion pathway, are elevated in PDO_CAF (Figure S12D, Supporting Information). Upon adding CAF, the chromatin landscapes of specific genes of PDO‐CAF were more similar to PDOX than to PDO (Figure S12E, Supporting Information). Hence, part of the differences in chromatin accessibility remodeling between PDO and PDOX was caused by interactions between tumor cells and CAF.

**Figure 3:**
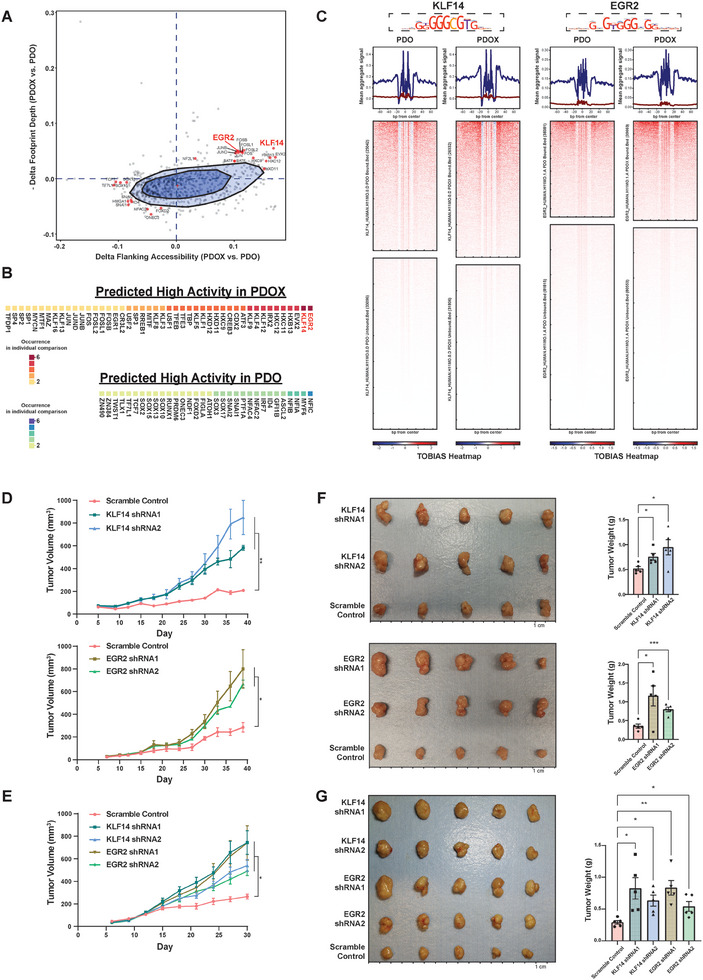
Transcription factor activities are affected by PDO→PDOX remodeling. A) BaGFoot analysis of PDOX versus PDO based on consensus peak set derived from each condition. The TFs predicted to be active in PDOX are in the first quadrant beyond the fence area with an extending factor of 1.5. The model‐specific TFs (also predicted to be active in PDOX in the comparison of PDOX versus PT, Table S5, Supporting Information), as well as EGR2 and KLF14, are highlighted. The TFs in the third quadrant beyond the fence area are predicted to be active in PDO. The model‐specific TFs (also predicted active in PDO in the comparison of PDO versus PT, Table S5, Supporting Information) are highlighted. B) The occurrence counts of high‐activity TFs in PDOX versus PDO from BaGFoot analysis for individual patient sets. C) TOBIAS footprint analysis of KLF14 (motif: KLF14_HUMAN.H11MO.0.D) and EGR2 (motif: EGR2_HUMAN.H11MO.1.A). The aggregated signals are summarized at the top; blue lines are from bound sites, and red lines are from unbound sites. The footprint heatmaps indicate the chromatin accessibility in the bound and unbound sites of KLF14 and EGR2 in PDO and PDOX. D,E): Tumor growth curves of PDOX106 (D) and PDOX187 (E) for KLF14 and EGR2 shRNA KD or the scrambled control. Error bars denote SEM of five replicates. p values were calculated based on repeat measurement one‐way ANOVA with Fisher's LSD test. *p < 0.05, **p < 0.01. F,G): Photos of xenograft tumors (left) and tumor weight comparisons (right) of PDOX106 (F) and PDOX187 (G) for KLF14 and EGR2 KD and the scramble control. The ruler of the tumor photo has 1‐cm intervals. Error bars in the tumor weight plots denote SEM of five replicates (mouse replicates are shown as scatter dots). p values were calculated based on ANOVA with post‐hoc. *p < 0.05, **p < 0.01, ***p < 0.001.

To investigate potential transcription factors that may regulate this process, we performed bivariate genomic footprinting (BaGFoot) analysis, which is based on the changes in the depth of TF footprint and TF motif‐flanking accessibility.^[^ ^33^ ^]^ Fifty‐seven TFs presented higher activities in PDOX, and 47 TFs exhibited higher activities in PDO (**Figure** **3**A, Table S5, Supporting Information). Several of these TFs were also identified when comparing PDO versus PT and PDOX versus PT, respectively (Figure S13A, Supporting Information), including EVX2, reported to be methylated in lung cancer,^[^ ^34^ ^]^ and SNAI1/2, involved in epithelial‐to‐mesenchymal transitions (EMT) and responsive to EGF.^[^ ^35^, ^36^ ^]^ These TF footprints were further analyzed by TOBIAS BINDetect^[^ ^37^ ^]^ (Figure S13B, Supporting Information).

We then performed BaGFoot analysis for each individual patient set (Figure S14A, Supporting Information). Notably, two TFs, Krüppel‐like family 14 (KLF14) and Early Growth Response 2 (EGR2), stood out (Figure 3B; Figure S14B, Supporting Information). TOBIAS footprint analysis indicates that KLF14 and EGR2 had deeper footprints in PDOX than PDO (Figure 3C; Figure S14C, Supporting Information), which was independently confirmed by the additional PDO‐PDOX sets (Figure S14D, Supporting Information). Moreover, PDOX have overall higher protein levels of KLF14 and EGR2 compared to corresponding PDO (Figure S14E, Supporting Information). Krüppel‐like family factors have been reported to regulate a multitude of cancer‐relevant processes.^[^ ^38^ ^]^ KLF14 precludes KRAS‐associated cell growth and transformation,^[^ ^38^, ^39^ ^]^ and loss of KLF14 can trigger PIK4‐mediated centrosome amplification to promote colon tumorigenesis.^[^ ^40^ ^]^ KLF14 has also been associated with high‐density lipoprotein cholesterol levels and metabolic syndrome.^[^ ^41^ ^]^ EGR2 might entail multifaceted roles in cancer. ALDH^positive^ tumor initiating cells have been shown to involve EGR2 upregulation.^[^ ^42^ ^]^ Meanwhile, EGR2 transactivates BNIP3L/BAK in PTEN‐induced apoptosis^[^ ^43^ ^]^ and exerts growth‐suppressive effects.^[^ ^44^ ^]^ Moreover, EGR2 binds ARF promoters and p16 to induce senescence.^[^ ^45^ ^]^ EGR2 malfunctions are also known to be related to cancer.^[^ ^46^, ^47^ ^]^

To assess their functions, we knocked down KLF14 and EGR2 in both PDO106 and PDO187 using lentiviral shRNA and injected the PDO into immune‐deficient NSG mice to form PDOX (Figure S15A, Supporting Information). For each gene, we used two shRNAs along with a scrambled shRNA as the control (Figure S15B–D, Supporting Information). In general, the silencing of KLF14 or EGR2 did not significantly alter the growth of the PDO in vitro (Figure S15E, Supporting Information). However, KLF14‐ and EGR2‐deficient PDOX grew faster than PDOX with scramble control shRNA (Figure 3D,E). We collected all xenograft tumors at the end of the experiments. For CRC106, the final tumor masses were 1.46‐ and 1.83‐fold higher in KLF14‐deficient PDOX and 3.26‐ and 2.25‐fold higher in EGR2‐deficient PDOX compared to the scrambled control group (Figure 3F). For CRC187, the final tumor masses of KLF14‐shRNA1, KLF14‐shRNA2, EGR2‐shRNA1, and EGR2‐shRNA2 were 2.84‐, 2.18‐, 2.87‐, and 1.90‐fold higher than control PDOX, respectively (Figure 3G). In addition, the differentially expressed genes in response to KLF14 or EGR2 silencing in PDOX were mostly regulated by the KLF14 or EGR2 (Table S6, Figure S15F,G, Supporting Information). Therefore, KLF14 and EGR2 binding activities were upregulated by the in vivo environment, and reducing KLF14 and EGR2 resulted in enhanced tumor growth.

**Figure 4:**
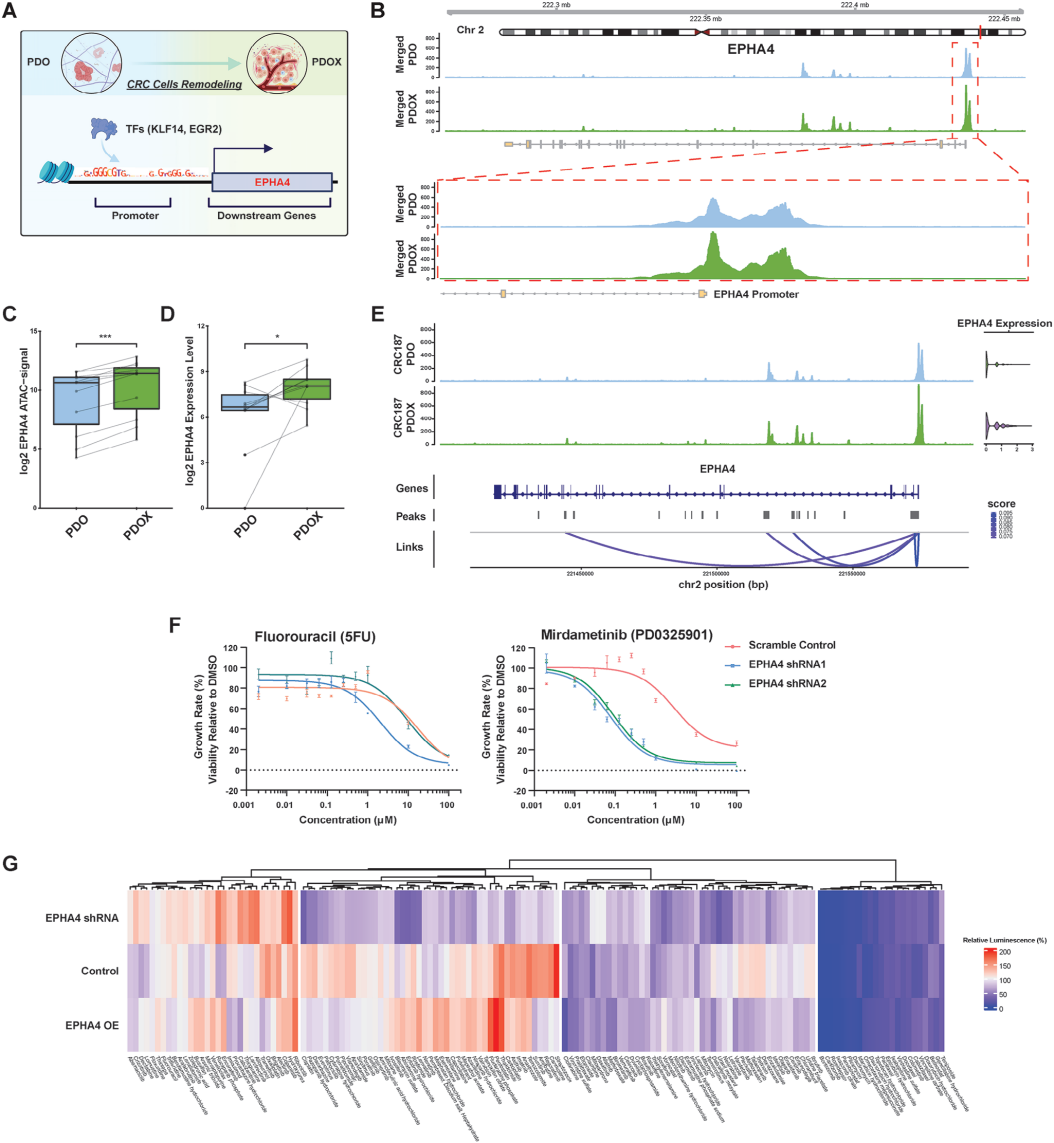
Downstream gene EPHA4 affects drug sensitivities. A) The illustration shows the epigenetic reprogramming of CRC cells from PDO in vitro to PDOX in vivo, in which the enhanced binding activities of TFs (KLF14 and EGR2) would lead to the higher expression of the downstream genes (EPHA4). B) ATAC‐seq signal track showing EPHA4 locus in PDO and PDOX. The exon locations are indicated in the gene map. The promoter areas of EPHA4 are circled and presented in the bottom panel. C) The boxplot reports cumulative ATAC‐seq signals in the EPHA4 region in paired sets of PDOX versus PDO. p values were calculated based on paired Student's t‐test. ***p < 0.001. D) The boxplot reports the EPHA4 expression levels based on mRNA‐seq in paired PDOX versus PDO. p values were calculated based on paired Student's t‐test. *p < 0.05. E) Peak–gene links for EPHA4 based on PDO and PDOX187 single cell multiome data showing the correlations between DNA accessibilities and EPHA4 expression. F) PDO106 growth rate dose‐response curves to Fluorouracil and Mirdametinib after knocking down EPHA4. Error bars denote SEM of four replicates. G) Heatmap of drug screen on PDO106 (control PDO, EPHA4 shRNA PDO, and EPHA4 overexpression [OE] PDO) showing the average relative luminescence (%) of three replicates for each compound and condition.

We then analyzed the genes downstream of KLF14 and EGR2 by integrating ATAC‐seq detected peaks and gene expression (**Figure** **4**A) and identified 24 genes associated with both EGR2 and KLF14 that are upregulated in PDOX (Table S7, Supporting Information). Among them, KLF4 and EPH Receptor A4 (EPHA4) were the top two candidates. Since KLF4 is a TF just like KLF14 and EGR2, we focused on EPHA4. EPHA4 has been reported to interact with fibroblast growth factor receptors (FGFRs). Specifically, EPHA4 can form a heteroreceptor complex with FGFR1^[^ ^48^ ^]^ or other FGF receptors (FGFR2, FGFR3, or FGFR4) via interaction between the juxtamembrane domain of FGFRs and N‐terminal portion of the tyrosine kinase domain of EPHA4,^[^ ^49^ ^]^ which influences FGFR‐mediated MAPK and AKT pathways.^[^ ^48^, ^49^, ^50^ ^]^ The KLF14 and EGR2 binding sites in the EPHA4 regulatory region were more accessible in PDOX than in PDO in all 11 cases, consistent with higher EPHA4 gene expression in PDOX (Figure 4B–D; Figure S16A, Supporting Information). We also observed the correlation between the expression levels of EGR2 and EPHA4 within the Cancer Genome Atlas (TCGA) dataset (Figure S16B, Supporting Information). Additionally, single cell multiomics data link noncoding accessible DNA elements to the expression of this gene.^[^ ^51^ ^]^ The elevated expression of EPHA4 in PDOX is associated with the collective regulation of the promoter and three enhancers, where the promoter has the highest correlation score (Figure 4E; Figure S16C, Supporting Information). Furthermore, ChIP‐qPCR confirmed that there is more EGR2 binding to the promoter region of EPHA4 in PDOX than in PDO (Figure S16D, Supporting Information, data of KLF14 is not showing since there is no ChIP‐grade antibody).

To examine whether KLF14‐ and EGR2‐mediated chromatin remodeling of EPHA4 affects drug sensitivity, we knocked down EPHA4 in the PDO (Figure S16E, Supporting Information) and then evaluated the responses to 5‐Fluorouracil (5FU, chemotherapy), Pemigatinib, Erdafitinib (FGFR inhibitors), and Mirdametinib (MEK inhibitor). Silencing of EPHA4 did not change PDO sensitivity to 5FU and had minor effects on FGFR inhibitors; however, silencing of EPHA4 sensitized PDO to Mirdametinib significantly (Figure 4F; S16F,G, Supporting Information). We further validated this finding by knocking out EPHA4 in PDO using CRISPR (Figure S16E,H, Supporting Information). Silencing of EPHA4 in vivo also has an effect on Mirdametinib responses (Figure S16I,J, Supporting Information). We observed a decrease in p‐ERK1/2 upon silencing of EPHA4 in PDO, whereas overexpressing EPHA4 resulted in an increase of p‐ERK1/2 (Figure S16K, Supporting Information), consistent with prior studies.^[^ ^50^, ^52^ ^]^ In addition, we noted a similar behavior in p‐MEK1/2 to that of p‐ERK1/2, potentially elucidating how EPHA4 could impact tumor sensitivity to the MEK inhibitor. In addition, a screen of 147 FDA‐approved anticancer compounds revealed that EPHA4 impacts PDO responses to a significant number of drugs (Figure 4G). Therefore, the differential binding of KLF14 and EGR2 and expression of downstream EPHA4 impact both tumor growth and drug sensitivity, suggesting that chromatin remodeling in different PDMC may interfere with their ability to predict therapeutic outcomes.

## 3 Discussion

PDMC are becoming the gold standard pre‐clinical models for therapeutic development and precision oncology. However, how tumor cells evolve in PDMC remain largely unclear. In this study, ATAC‐seq, supplemented by RNA‐seq, provided a comprehensive picture of the changing chromatin accessibility landscape in PDMC from original PT. Notably, all PDMC are separated from PT along the first PC axis, whereas in vitro and in vivo PDMC are separated along the second axis. The observation that PDOX is more like PDX than PDO suggests the possibility that the PDMC environment is a potential force influencing chromatin remodeling in tumor cells that can be partially recapitulated by co‐culturing PDO with CAF. Besides CAF, other non‐malignant cells in the tumor microenvironment may also influence CRC epigenetic regulation in PDOX models. For example, immune cells are known to be important in shaping tumor cell evolution, but the NSG xenograft models in this study possessed limited and dysfunctional immune cells. Humanized PDX models^[^ ^53^ ^]^ might offer further insights into how immune cells may modulate CRC epigenetics. CRC of different genomic subtypes seem to largely undergo the two chromatin remodeling axes, although genetic diversity, loss of rare genetic clones, or *de novo* genomic alterations during PDMC passaging likely contribute to individual chromatin remodeling.

As part of the NCI patient‐derived model consortium, we and other researchers have consistently observed that PDOX are generally faster to develop, with higher success rates, than PDX. The difference is more significant for cancer types for which the success rates of conventional PDX are low.^[^ ^54^, ^55^ ^]^ However, we do not have a complete understanding of the tradeoffs. The fact that PDOX is similar to PDX in terms of chromatin accessibility suggests that PDOX may provide a reasonable alternative to PDX for certain applications.

A comparison of PDO and PDOX further highlighted the regulatory elements that respond differently to in vitro versus in vivo environments. BaGFoot and TOBIAS Footprinting analyses revealed TFs that differentially bind to open chromatins in the two models. Among these TFs, KLF14 and EGR2 have enriched motif‐binding footprints in PDOX. Silencing of KLF14 and EGR2 led to enhanced CRC growth in PDOX. This suggests that the mouse stroma can elicit tumor suppressors that slow down the tumor growth, which may partially explain why PDX tend to grow slower than PDO. In addition, the differential expressions of downstream genes like EPHA4 may alter sensitivity and resistance to drugs such as targeted therapy. Therefore, chromatin remodeling of different PDMC may interfere with their ability to predict therapeutic outcomes and needs to be more carefully examined. This work provides a resource for the PDMC community to examine the differences among CRC PDMC models, and similar studies in other cancer types are likely to be informative.

## 4 Experimental Section

### Patient Samples

The CRC specimens were collected at Duke University Hospital through the Duke BRPC, which is part of the National Cancer Institute's Cooperative Human Tissue Network. The study was reviewed and approved by the Duke Institutional Review Board (Pro000089222). Informed consent was obtained from all participants. The mutation data were obtained from clinical colon hotspot panels or WES analyses. Each surgically removed tumor specimen was minced and then split for three purposes: 1) sequencing as the PT samples, 2) deriving the matched PDO (described in the following method), 3) deriving the matched PDX (described in the following method).

### Tumor Isolation and Patient‐Derived Organoid Culture

The PDO development and cultivation methods were modified based on the previously published method.^[^ ^1^, ^21^, ^56^ ^]^ Briefly, the CRC specimen was minced and incubated in digestion buffer (HBSS with 1 mg mL^−1^ collagenase from Clostridium Histolyticum, 0.1 mg mL^−1^ DNase I, 3 mm calcium chloride, 100 µg mL^−1^ primocin, and 10 µm Y‐27632) at 37 °C for 60 min in the orbital shaker. Large fragments in the mixture were removed by passing through a 100‐µm cell strainer after the incubation. The cells were centrifuged and washed twice with PBS. Cells were counted, embedded in ice‐cold Matrigel, and inoculated in 24‐well plates. After at least 15 min at 37 °C, Matrigel was polymerized. The CRC culture medium (Advanced DMEM/F12 with 2 mm GlutaMAX, 10 mm HEPES, 100 U mL^−1^ Penicillin and Streptomycin, 1X B27, 1.25 mm n‐Acetyl Cysteine, 10 nm Gastrin I, 500 nm A‐83‐01, 3 µm SB202190, 100 ng mL^−1^ Recombinant Human R‐Spondin, 100 ng mL^−1^ Recombinant Human Noggin, 50 ng mL^−1^ Recombinant Human EGF, 10 nm Prostaglandin E2, 100 µg mL^−1^ Primocin, and 10 mm Nicotinamide) was added and refreshed every two to three days.

For the PDO that successfully grew and derived, the organoids were passed every 1–2 weeks based on their growth rates. To passage the organoids, the CRC culture medium was removed, and the Matrigel dome was degraded with PBS. The mixture of PBS with Matrigel‐organoids was collected and centrifuged at 1500 RPM for 5 min, and the PBS was removed. TrypLE express enzyme (#12604013, Gibco) was added, and organoids were incubated at 37 °C for 10 min. After the addition of PBS, the cells were centrifuged, rewashed with PBS, and seeded with Matrigel in a 24‐well plate. Culture medium was added after Matrigel polymerization. The bright‐field images were taken by a Leica DMIL LED Fluorescent Microscope. Part of the established PDO was dissociated and then cryopreserved for sequencing using frozen medium (70% FBS + 20% culture medium + 10% DMSO) in liquid nitrogen. For CAF‐PDO coculture experiments, the CAF was isolated from PDOX and cocultured them with the matched PDO at 1:2 ratio in Matrigel for 7 days. Then, the CRC cells from PDO were harvested by removing the CAF. The nuclei of CRC cells were extracted for ATAC‐seq.

### PDO H&E‐Staining and Immunolabeling Fluorescence Staining

PDO were fixed and embedded in paraffin per a modified Trevigen, Inc. protocol.^[^ ^57^ ^]^ First, Matrigel domes with PDO were washed with PBS and then fixed with 5 mL of 2% paraformaldehyde (PFA) + 0.1% glutaraldehyde (GA) in PBS at room temperature for 30 min. After washing with PBS, the domes were taken to 20% sucrose and left overnight at 4 °C until the domes sank to the bottom. Next, the solution was changed to 70% ethanol, and the domes with PDO were embedded with paraffin for sectioning and H&E‐staining in the Duke Pathology Research Histology Lab.

The CRC identity of PDO was confirmed by the maintained protein expression of CDX2 and CK20 (Figure 1C; Figure S1A,B, Supporting Information). A publsihed PDO 3D imaging protocol was followed.^[^ ^58^ ^]^ Briefly, the organoids were recovered from Matrigel using the ice‐cold cell recovery solution (Corning) and then fixed with 4% PFA at 4 °C for 45 min. After blocking, PDO were cultured with primary and then secondary antibodies for immunolabeling. Fructose–glycerol clearing solution was used for imaging the organoids under the Leica SP5 inverted confocal microscope. The following antibodies were used for immune‐fluorescence staining: CDX2 (12306S, Cell Signaling Technology), 1:200; CK20 (60183‐1‐IG, Proteintech), 1:100; Anti‐Rb IgG‐488 (ab150061, Abcam), 1:500; Anti‐Ms IgG‐594 (ab150112, Abcam) 1:500.

### Xenograft Development

All animal experiments were approved by the Duke Animal Care and Use Program (IACUC) following the A235‐18‐10 and A112‐18‐05 protocols. PDX were developed as described previously.^[^ ^59^, ^60^ ^]^ The CRC tissue was minced and resuspended in 100 µL of PBS and then injected subcutaneously (s.c.) into the flanks of NOD‐SCID IL2Rgamma^null^ (NSG, JAX005557) mice. When the tumor reached a volume of 1000 mm^3^ [the tumor volume (V) was determined as *V*  = *width* ^2^  × *length* ÷ 2], it was harvested, minced, and resuspended in PBS before s.c. injection into the flanks of other NSG mice. Part of the tumor tissue from PDX was cryopreserved in 70% FBS + 20% DMEM + 10% DMSO for future passages. Organoid xenografts (PDOX) were also developed using a modified version of the published protocol.^[^ ^61^ ^]^ PDO were harvested by removing Matrigel with TrypLE express enzyme, counted, and resuspended in 50% Matrigel/PBS solution. Cells (1 × 10^6^) were injected s.c. into the flanks of NSG mice. The harvested xenograft tumors were dissociated into single cells using the Tumor Dissociation Kit (Miltenyi Biotech, # 130‐095‐929), and then they were cryopreserved before sequencing using frozen medium (70% FBS + 20% culture medium + 10% DMSO) in liquid nitrogen.

The xenograft models maintained histopathological characteristics similar to the patient tumors from which they were derived, and they were histologically confirmed to be CRC by H&E staining and IHC expression of CDX2+/CK20+/CK7‐ (Figure 1C; Figure S1C,D, Supporting Information). For xenograft staining, tumors were harvested, fixed in 10% neutral buffered formalin, and then embedded in paraffin. Sections were subjected to H&E as well as immunohistochemical staining in the Duke Pathology Research Histology Lab. The following antibodies were used for immune‐fluorescence staining: CDX2 (PA0375, Leica Biosystems); CK20 (PA0022, Leica Biosystems); CK7 (ab68459, Abcam).

### shRNA and gRNA Lentivirus Transduction of PDO

The KLF14, EGR2, and EPHA4 lentivirus constructs were obtained from Sigma Mission shRNA (KLF14 shRNA1: SHCLNG‐NM_138 693, TRCN0000107420; KLF14 shRNA2: SHCLNG‐NM_138 693, TRCN0000107424; EGR2 shRNA1: SHCLNG‐NM_000399, TRCN0000013840; EGR2 shRNA2: SHCLNG‐NM_000399, TRCN0000013841; EPHA4 shRNA1: SHCLNG‐NM_0 0 4438, TRCN0000010165; EPHA4, shRNA2: SHCLNG‐NM_0 0 4438, TRCN0000332976; Scramble Control: pLKO scramble shRNA puro, Addgene #1864). The CRISPR knock‐out plasmids were obtained from GenScript USA, Inc (eSpCas9‐LentiCRISPR v2). The gRNA sequence for EPHA4 is CAGTTCGTAGCCAGTTATTC. The lentiviral vectors were co‐transfected with packaging and envelope plasmids (pCMVR8.74, Addgene #22 036; pCMV‐VSV‐G, Addgene #8454) into 293T cells. The viral supernatant was collected and concentrated using Lenti‐X Concentrator (TaKaRa, 631 232) 48 h after transfection. The transduction process was from a modified published protocol.^[^ ^62^ ^]^ Briefly, PDO were harvested and resuspended in 500 µL CRC culture medium. Next, the organoids solution was mixed with 500 µL viral particles and 5 µL TransDux (System Biosciences, LV850A‐1). The mixture was transferred to a 12‐well plate and centrifuged at ≈30 °C at 600 g for 60 min. Then, the organoids‐virus mixture was incubated for another 3 h at 37 °C before Matrigel embedding and culturing in the CRC culture medium. On day 3 after transduction, puromycin (5 µg mL^−1^ for PDO106 and 2 µg mL^−1^ for PDO187) was added to the medium to select the cells carrying constructs of interest. After 2 weeks of selection, the knockdown efficiencies of KLF14, EGR2, and EPHA4 were evaluated by TaqMan qPCR using relative gene expression (ΔΔC_T_ Method), and the statistical significance of each experiment was assessed based on ANOVA using ΔCt.^[^ ^63^ ^]^ Briefly, total RNA was extracted from PDO using the Norgen single cell RNA purification kit (Norgen, 51 800). The TagMan primers and probes were obtained from Thermo Fisher, and ACTB was leveraged as the endogenous control (KLF14: Hs00370951_s1; EGR2: Hs00166165_m1; EPHA4:  Hs00953178_m1; ACTB: Hs01060665‐g1). The growth of the PDO was monitored utilizing the ImageXpress Pico platform (Molecular Devices, LLC). The CRC cells were cultured in 96‐well plates and labeled by anti‐EPCAM‐Alexa Fluor 647 (1:1000 in CRC medium, Abcam #AB237396) prior to imaging. The changes in the positive areas (indicated by Alexa Fluor 647, calculated by CellReporterXpress) were measured for CRC growth. The expression levels of proteins were evaluated by western blots as previously described^[^ ^64^ ^]^ using anti‐KLF14 (Invitrogen, PA5‐100880), anti‐EGR2 (Proteintech, 13491‐1‐AP), anti‐EPHA4 (BD biosciences, Cat#610 471), anti‐phospho‐MEK1/2 (Ser217/221) (Cell Signaling Technology, 9154T), anti‐phospho‐p44/42 MAPK (Erk1/2) (Thr202/Tyr204) (Cell Signaling Technology, 4370T), anti‐p44/42 MAPK (Erk1/2) (Cell Signaling Technology, 4695T), and anti‐beta‐Actin (Cell Signaling Technology, 4970S) antibodies.

### ChIP qPCR

ChIP qPCR was performed using the commercial kit (SimpleChIP Enzymatic Chromatin IP Kit (Magnetic Beads) #9003, Cell Signaling Technology), and the protocol of the kit was followed. Briefly, PDO or homogenized PDOX samples were cross‐linked by formaldehyde and the nuclei were extracted then treated with Micrococcal Nuclease to generate digested chromatin (150‐900 bp). The digested chromatin was incubated with anti‐EGR2 (Invitrogen, MA5‐38053) or normal rabbit IgG (Cell Signaling Technology, #2729) overnight at 4 °C. Two percent of the input samples (without antibody nor IgG) were set aside until chromatin DNA elution. Protein G magnetic beads were utilized to purify the antibody‐chromatin IP complex. Chromatin DNA (including the 2% input sample) was then eluted and purified. Real‐time quantitative PCR was performed using the primer set targeting the promoter region of EPHA4 (forward 5′‐CCTTTGGTTACAAACTTGGACAG and reverse 5′‐GGGAGCCCAGTGTGAATG). The percentage inputs of both anti‐EGR2 and IgG control were calculated and reported.

### Compound Sensitivity Testing

PDO were seeded into 96‐well plates at 3000 viable cells per well with 10 µL Matrigel. Next, 100 µL CRC culture medium containing RealTime‐Glo MT Cell Viability Assay (Promega G9711) and compounds of a 10‐point dose curve along with DMSO control were added. The cell viability at 0 h for each well was measured by RealTime‐Glo MT Cell Viability Assay for growth rate correction. The organoids were treated (drug or DMSO) for 5 days prior to assessing for viability. The PDO viabilities were determined by the CellTiter‐Glo 3D Cell Viability Assay (Promega, G9683). Briefly, 100 µL CellTiter‐Glo 3D was added to each well, and the plate was shaken for 5 min followed by a 20 min incubation at room temperature. The luminescence was measured by the plate reader (Varioskan LUX). Each dose point was normalized to the DMSO control for relative viability. The growth‐rate dose response curve was fit to a 3‐parameter sigmoidal curve using GraphPad Prism 9. The compounds used for the testing were 5‐Fluorouracil (Millipore Sigma, F6627‐5G), Mirdametinib (PD0325901) (Millipore Sigma, PZ0162‐25MG), Pemigatinib (Selleckchem, Catalog No.S0088), and Erdafitinib (Selleckchem, Catalog No.S8401). The high throughput drug screen was performed by the Duke Functional Genomics Core. Briefly, the NIH‐approved oncology drug set IX (plate 4891 and 4892, including 147 compounds) was used for the screen. The EPHA4 overexpression (OE) PDO were transduced with EPHA4 ORF lentivirus (EPHA4_OHu24718C_pGenlenti, GenScript). The EPHA4 shRNA1 lentivirus were used for EPHA4 knocking down PDO. The PDO were exposed to the compounds (1 µm each; *N* = 3 for each compound and each PDO condition) for three days. The viabilities were then determined by the CellTiter‐Glo 3D Cell Viability Assay. The luminescence for each drug was normalized to the DMSO‐treated PDO. The heatmap was generated by the R package ComplexHeatmap with the K‐nearest‐neighbor clustering km = 4.

### In Vivo PDOX Growth Measurements

PDO with distinct types of shRNAs were harvested, counted, and resuspended in 50% Matrigel PBS solution at a concentration of 10^7^ cells mL^−1^. PDO solution (100 µL, 1 million cells) was injected s.c. into the flanks of NSG mice using a 23‐G needle. The tumor length and width were measured with a 0.01‐mm vernier caliper every three days. The tumor volume (V) was determined as *V*  = *width* ^2^  × *length* ÷ 2. At the end of the study, the tumors were harvested and weighed. Five mice for each type of PDO were used, and five mice were used for the scramble control group. The Mirdametinib treatment was started when the tumor reached a volume of 200 mm^3^. Mirdametinib was administrated by oral gavage at 20 mg kg^−1^ daily for 24 days.

### ATAC‐seq and mRNA‐seq Library Preparation and Sequencing

The mouse cell components were removed from the xenograft samples and dead cells from all DMSO‐preserved samples before preparing the sequencing libraries. Briefly, cells were recovered from the DMSO cryopreservation, washed with PBS, and spun down. The supernatant was removed and resuspended in 50 µL mouse depletion cocktail (for the xenografts samples, Miltenyi Biotech, #130‐104‐694) + 100 µL dead cell removal microbeads (Miltenyi Biotech, #130‐090‐101) + 60 µL binding buffer and incubated 15 min at room temperature. The mixture was passed through an LS column (Miltenyi Biotech, #130‐042‐401), and the LS column was washed three times with 2 mL binding buffer. The live human cells were in the flow‐through and ready for the next step. The Omni‐ATAC protocol^[^ ^26^ ^]^ was utilized for ATAC library preparations. The nuclei were extracted from samples (PT, PDO, PDX, and PDOX). For each sample type in one PT‐PDO‐PDX/PDOX set (e.g., PDOX106), three replicates were included (xenografts were from three mice). Intact nuclei (5 × 10^4^) were used for each replicate. Transposase, digitonin, and Tween‐20 were added, and the mixture was incubated at 37 °C for 30 min. The transposed fragments were amplified, with the number of cycles determined by qPCR for each sample. The libraries that passed QC were sequenced by Illumina HiSeq 4000 PE150 bp. The RNA was extracted using the Norgen single cell RNA purification kit (Norgen, 51 800). The mRNA‐seq libraries were made by Novogene and sequenced by Illumina HiSeq 4000 PE150 bp. The Chromium Next GEM single cell Multiome kit (10X Genomics) was utilized for single cell multiome ATAC + gene expression library preparation. Briefly, the CRC187 PT‐PDMC set with patient, PDO, PDOX, and PDX samples was used for 10X single cell sequencing. Eight thousand nuclei from each sample were extracted and transposed. Then, the GEMs were generated using the Chromium Next GEM Chip J, and barcodes were added. After cleanup and pre‐amplification, the single cell ATAC libraries and the single cell gene expression libraries were constructed following the 10X protocol (CG000338 Rev B). The libraries were sequenced using NovaSeq 6000 S1 PE100 bp.

### ATAC‐seq and mRNA‐seq Data Processing

ATAC‐seq data was processed using the pipeline developed for ENCODE (https://github.com/ENCODE‐DCC/atac‐seq‐pipeline) to perform quality control, filtering of low‐quality reads and PCR duplicates, analysis of reproducibility, reference genome alignment, peaks calling, and fold‐enrichment or p‐value signal tracks generation. Reads were aligned to reference genome hg19 using Bowtie2.^[^ ^65^ ^]^ Duplicate and mitochondrial reads were removed, and peaks were called by MACS2.^[^ ^66^ ^]^ RNA‐seq raw sequencing reads were quality checked using Fastqc and summarized with MultiQC.^[^ ^67^ ^]^ Transcripts were aligned to reference genome hg19 using Hisat2 and quantified by HTSeq.^[^ ^68^, ^69^ ^]^

### Differential Peak Calling and Annotation

Differential peaks were identified using DiffBind with a minimum of three biological replicates.^[^ ^27^ ^]^ The fold‐changes of peaks were evaluated by the DESEQ2 method in the DiffBind package. Peaks with p‐value < = 0.05 and |log2FC| > = 1 were considered differentially accessible. Peaks annotation was performed using the findMotifsGenome.pl command in the HOMER2 software based on the nearest TSS.^[^ ^70^ ^]^ The R package ChIPseeker was also used to annotate peaks and visualization.^[^ ^71^ ^]^ Gene Ontology was performed by the R package clusterProfiler.^[^ ^72^ ^]^ For mRNA‐seq, Deseq2 was used for differential expression analyses. Genes differentially expressed were classified by p‐value < = 0.05 and log2FC > = 1.^[^ ^73^ ^]^

### ATAC Track Visualization and Correlation

Bam files from each ATAC library were used as input for deeptool2 bamCoverage command to generate the bigwig files.^[^ ^74^ ^]^ The bigwig files were visualized by the R package Gviz. The correlations between different bam files were performed using the deeptool2 multiBamsummary command and the resulting matrix was plotted by the plotCorrelation –whatToPlot scatterplot command.

### Sequencing Data Visualization

Circos plots were generated based on the differential or consensus peaks generated by Diffbind using the R package circlize.^[^ ^75^ ^]^ Briefly, circos.genomicDensity function was used to visualize the genomic density of the genome.

Heatmaps, PCA plots, and MA plots were generated using the built‐in Diffbind methods. The heatmap for clustering was generated based on the 1000 peaks with the highest variance. The PCA plot was generated based on the DBA_SCORE_RPKM of each replicate. The heatmap for chromatin accessibility around a TSS was generated using deepTools.^[^ ^74^ ^]^

The Pearson correlation plots between genes of interest in TCGA COAD patients were generated using the online tool GEPIA2.^[^ ^76^ ^]^ The expression level of each gene in the RNA‐seq was TPM normalized.

### ATAC Differential Occupancy and Pathway Analysis

The consensus peak set for the PDMC models and patient ATAC‐seq results were generated by the R package Diffbind. In short, a count matrix was generated for the combined consensus peak set, and the counts were normalized with DBA_NORM_NATIVE and DBA_LIBSIZE_PEAKREADS options. Three contrasts: 1) PDO versus Patient, 2) PDOX versus Patient, and 3) PDX versus Patient were generated and used as input for dba.analyze with method = DBA_DESEQ2. The resulting DE peaks from all three contrasts were summarized into a data frame, and K‐means clustering with centers = 4 was performed to cluster the peaks into four different clusters. The clustered peaks were then visualized by the R package ComplexHeatmap.^[^ ^77^ ^]^ The shared gain and loss of peaks from different comparisons were then subjected to GREAT for pathway analysis.^[^ ^78^ ^]^

### 10X Single‐Cell Analysis

The Illumina sequenced 10x single‐cell multiome libraries were subjected to the 10x Cell‐Ranger‐Arc pipeline with default steps. Briefly, sequence data was demultiplexed into single‐cell ATAC results and RNA results by running the cellranger‐arc mkfastq command. Single cell feature counts for both ATAC and RNA results were then generated by the cellranger‐arc count command, with GRCh38 as a reference genome. Samples sequenced from different sequencing lanes were aggregated by the cellranger‐arc aggr command. The resulting count matrices for each PDMC and patient model were then treated as inputs for downstream analysis. The expression matrices were also subjected to SoupX to remove any predicted ambient RNA contamination with default parameters.^[^ ^79^ ^]^ Seurat objects were then created from the resulting Gene expression matrix for further aggregation and population identification.^[^ ^80^, ^81^ ^]^ The filtering scheme were: nCount_ATAC < 100 000 & nCount_ATAC > 1000 (200 for PT); nCount_RNA < 100 000 (30 000 for PT) & nCount_RNA > 1000 (500 for PDX, 200 for PT); nFeature_RNA > 1000 (500 for PDX, 100 for PT) & nFeature_RNA < 10 000; nucleosome_signal < 2; TSS.enrichment > 1; percent.mt < 30 (25 for PDX, 10 for PT). Peaks and gene linking were performed by the R package Signac. The analysis was performed by following the Joint RNA and ATAC analysis: 10x multiomic vignettes. The chromatin accessible regions and the gene expression levels were linked using the LinkPeaks function. The loom file was created using the velocyto command line tool based on the 10X multiomic output data. The RNA‐velocity analysis was performed using the scVelo package. In short, the Seurat object was output to metadata table, expression count matrix, dimensionality reduction matrix, and gene names and then manually converted into scVelo input files. The RNA velocity was computed, and the paga velocity‐graph was generated using the default parameters.

### Transcription Factor Analysis

BaGFoot Analysis was performed following the previously described methods.^[^ ^33^ ^]^ The hg19 genome was used as the reference. Aligned and filtered ATAC‐seq peaks from each biological replicate were pooled to create a consensus file for each condition and used for pairwise BaGFoot analysis. The outer polygon fence was created by inflating the bag geometrically by a factor of 1.5.

Footprint analysis was performed using the TOBIAS BINDetect tool.^[^ ^37^ ^]^ The meme file was downloaded from the HOCOMOCO database. Correction of Tn5 insertion bias, calculation of footprint scores within regulatory regions, estimation of bound/unbound transcription factor binding sites, and visualization of footprints within and across different conditions were performed using default parameters.

### Identification of Downstream Genes for KLF14 and EGR2

In the ATAC‐seq for PDOX versus PDO, the differentially enriched peaks were annotated to the nearest transcription start site and denoted as ATAC‐seq DE genes. The PDOX ATAC‐seq and RNA‐seq DE genes were thereby inner joined to find the PDOX ATAC+RNA‐seq enriched gene set. The downstream genes regulated by KLF14 and EGR2 were identified by using the R package tftargets. Table S7 (Supporting Information) was generated by taking the intersection of the KLF14 and EGR2 downstream genes from the Marbach 2016 dataset^[^ ^82^ ^]^ with the PDOX ATAC+RNA‐seq enriched gene set.

### Whole‐Exome Sequencing Analysis

All WES samples were processed using the same pipeline. Briefly, the raw fastq files were trimmed of adapters and low‐quality bases by fastp version 0.20.1,^[^ ^1^ ^]^ then mapped to the human reference genome (GRCh37) using BWA‐mem version 0.7.17^[^ ^2^ ^]^ in paired‐end mode. SAMtools version 1.9^[^ ^3^ ^]^ and Picard version 2.25.7 (http://broadinstitute.github.io/picard/) were utilized for sorting, merging, and removing duplication for the BAM files. The somatic mutations were called using MuTect2 (GATK version 4.2.5.0,^[^ ^4^ ^]^). Specifically, flags were included for “‐nct 1 ‐rf DuplicateRead ‐rf FailsVendorQualityCheck ‐rf NotPrimaryAlignment ‐rf BadMate ‐rf MappingQualityUnavailable ‐rf UnmappedRead ‐rf BadCigar” for MuTect2. A publicly available list of variants observed in a pool of normal DNA exome sequencing was used as a required input (https://console.cloud.google.com/storage/browser/details/gatk‐best‐practices/somatic‐b37/Mutect2‐exome‐panel.vcf, the GATK resource). The somatic mutations with suboptimal quality scores were removed. The remaining variants were annotated by SnpEff version 5.1.^[^ ^5^ ^]^ An exome‐capture target DNA sequence in a BED file was used to call mutations on WES data. The high‐confidence outputs or SNVs flagged as “PASS” in the resulting VCF files were applied to our downstream analysis. Seven Bridges' pipeline for NGS‐based copy number variation (CNV) detection (CNVkit Tumor‐Only CNV Calling with GATK HaplotypeCaller) was applied to detect CNV. CNVkit version 0.9.6^[^ ^6^ ^]^ and GATK version 4.2.5.0^[^ ^4^ ^]^ were used for the CNV calling and filtering in the pipeline. Any bins with log2 copy ratio lower than − 15 were considered artifacts and removed. For the CNV arguments ‐dbSNP, the dbsnp_137.b37.vcf was used, which was downloaded from the GATK Resource Bundle, as required inputs.

### Statistical Analysis

DiffBind and DESeq2 were used for the differential analyses of ATAC‐seq data, and DESeq2 was used for mRNA‐seq. Animal data were expressed as mean ± standard error of the mean (SEM) of at least three biological replicates. The number of biological replicates is denoted in the figure legends. Paired Student's t‐test or ANOVA were used for comparisons, with p‐value < = 0.05 considered to be significant. Mice were randomly allocated to experimental groups.

### Inclusion and Ethics statement

The human colorectal cancer specimens were collected through the Duke BioRepository & Precision Pathology Center, with informed consent obtained from all participants. The study was approved by the Duke Institutional Review Board (Pro000089222). All animal experiments on xenografts were approved by the Duke Animal Care and Use Program (IACUC) following the A235‐18‐10 and A112‐18‐05 protocols.

## Conflict of Interest

H.C.’s full disclosure is given at https://www.uu.nl/staff/JCClevers/. H.C. is inventer of several patents related to organoid technology, cofounder of Xilis Inc. and currently an employee of Roche, Basel. X.S. and D.H. are cofounders of Xilis Inc.

## Author Contributions

K.X. and E.W. contributed equally to the work. X.S., D.H., and K.X. initiated the idea and project. K.X., E.W., D.H., and X.S. designed the experiments. S.K., G.C., and X.W. advised on experimental designs and data analysis. E.W., K.X., A.D., C.J., and N.G. analyzed the data. K.X. performed the experiments with the assistance of M.N., G.S., E.W., and C.H. D.H., G.R., S.L., and S.J.M. facilitated patient tumor samples and medical data acquisition. D.H., G.R., P.S., and Y.K. developed PDX models. K.K. and H.C. provided support for the development of PDO. K.X., J.M., and M.N. established PDO and PDOX models and performed related experiments with the assistance of G.S., Z.W., Q.H., C.H., and P.R. K.X. prepared sequencing libraries with the assistance of G.S., L.W., R.X., and J.B. S.D. coordinated the Illumina sequencing. K.X., E.W., S.B., and X.S. wrote the manuscript.

## Supporting information

Supporting Information

Supplemental Table 3

Supplemental Table 4

Supplemental Table 5

Supplemental Table 6

Supplemental Table 7

## Data Availability

The data that support the findings of this study are openly available in Gene Expression Omnibus at GSE184482, reference number 184482.
