# Supplementary material for: Chromatin Remodeling in Patient‐Derived Colorectal Cancer Models
Source: Adv Sci (Weinh). 2024 Feb 21;11(16):2303379. doi: 10.1002/advs.202303379 (PMC11040356; doi:10.1002/advs.202303379)
Supplement: Supplementary file 1 — Supporting Information [file ADVS-11-2303379-s002.pdf]

ADVANCED SCIENCE
Open Access

Supporting Information

for *Adv. Sci.*, DOI 10.1002/advs.202303379

Chromatin Remodeling in Patient-Derived Colorectal Cancer Models

Kun Xiang, Ergang Wang, John Mantyh, Gabrielle Rupprecht, Marcos Negrete, Golshid Sanati, Carolyn Hsu, Peggy Randon, Anders Dohlman, Kai Kretzschmar, Shree Bose, Nicholas Giroux, Shengli Ding, Lihua Wang, Jorge Prado Balcazar, Qiang Huang, Pasupathi Sundaramoorthy, Rui Xi, Shannon Jones McCall, Zhaohui Wang, Chongming Jiang, Yubin Kang, Scott Kopetz, Gregory E. Crawford, Steven M. Lipkin, Xiao-Fan Wang, Hans Clevers, David Hsu* and Xiling Shen**

Figure S1

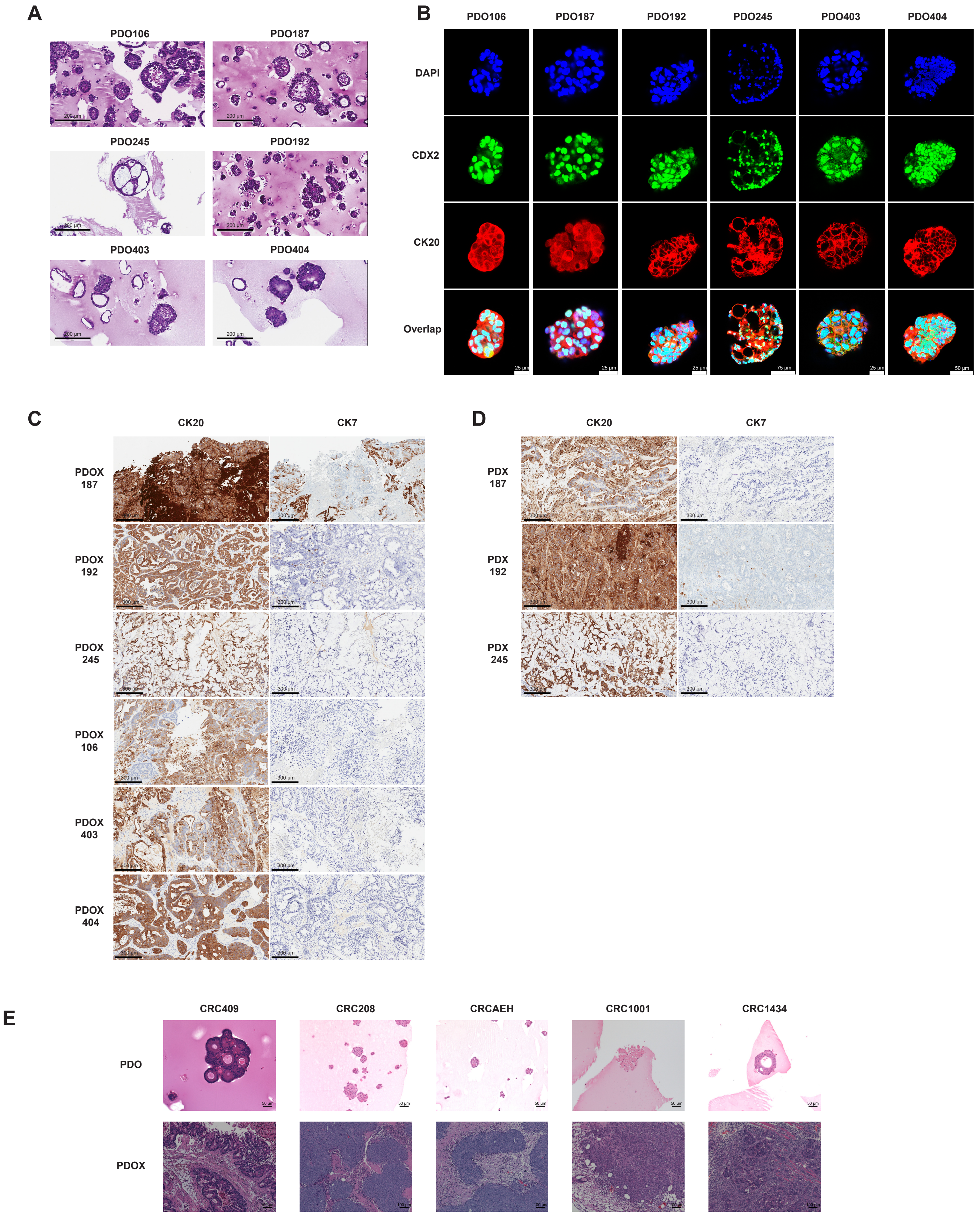

A
PDO106
PDO187
PDO245
PDO192
PDO403
PDO404
200 μm
B
PDO106
PDO187
PDO192
PDO245
PDO403
PDO404
DAPI
CDX2
CK20
Overlap
25 μm
75 μm
50 μm
C
CK20
CK7
PDOX 187
PDOX 192
PDOX 245
PDOX 106
PDOX 403
PDOX 404
300 μm
D
CK20
CK7
PDX 187
PDX 192
PDX 245
300 μm
E
CRC409
CRC208
CRCAEH
CRC1001
CRC1434
PDO
PDOX
50 μm

Figure S1 Histological staining of patient and PDMC.

(A) H&E-staining of paraffin-embedded PDO. Scale bars, 200 µm.

(B) High resolution whole-mount confocal image of PDO immunolabeled for CDX2 (green) and CK20 (red). DAPI is labeled in blue. The overlapping images are also shown in Figure 1C. Scale bars, 75 µm for PDO245, 50 µm for PDO404, and 25 µm for the rest.

(C) IHC staining for CK20 (left) and CK7 (right) of PDOX. Scale bars, 300 µm.

(D) IHC staining for CK20 (left) and CK7 (right) of PDX. Scale bars, 300 µm.

(E) H&E-staining of the additional PDO-PDOX validation sets.

Figure S2

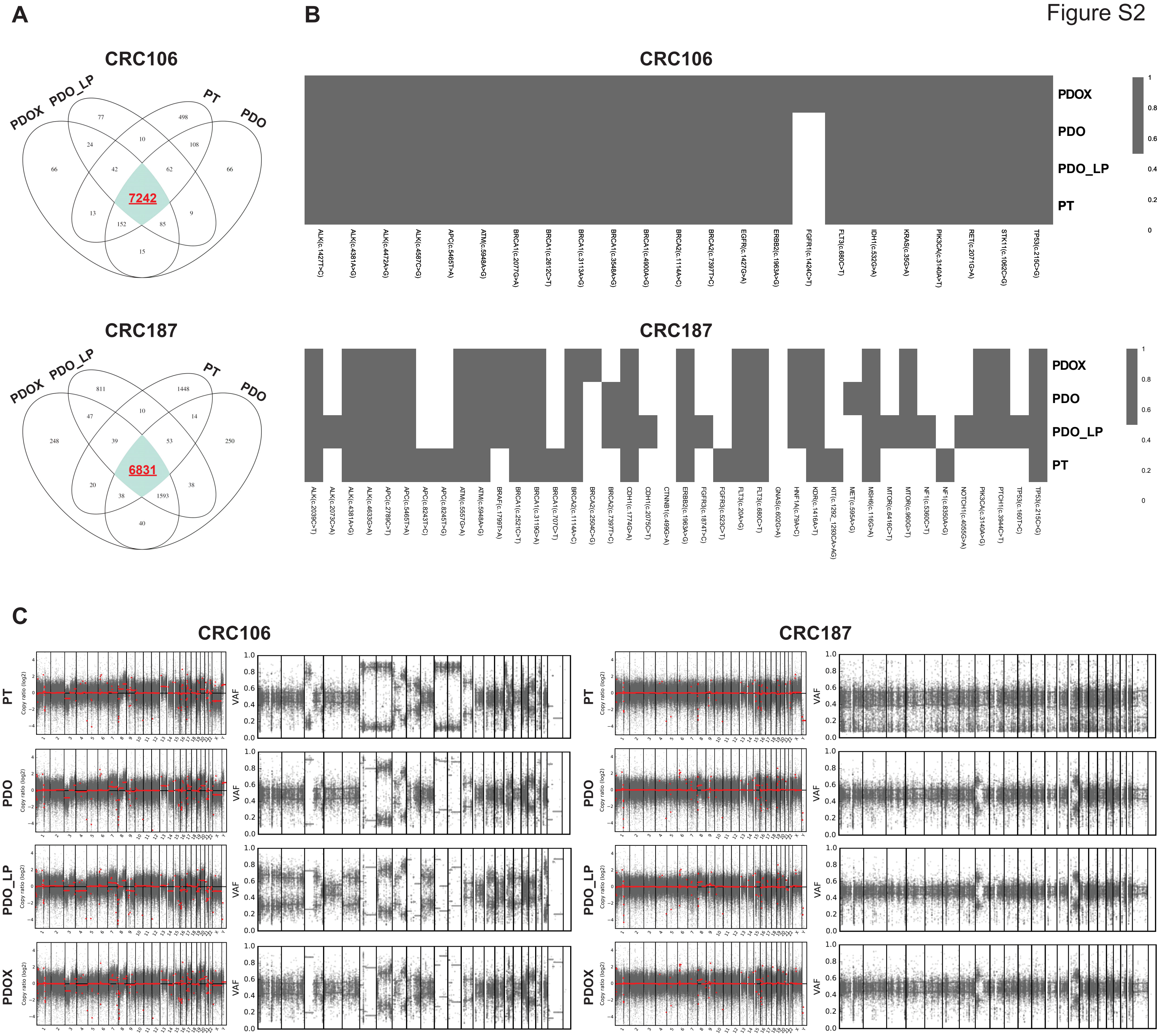
A
B
C
CRC106
PDOX
PDO_LP
PT
PDO
77
498
24
10
108
66
42
62
66
7242
13
9
152
85
15
CRC187
811
1448
47
14
248
39
53
250
6831
20
38
1593
38
40
ALK(c.1427T>C)
ALK(c.4381A>G)
ALK(c.4472A>G)
ALK(c.4587C>G)
APC(c.5465T>A)
ATM(c.5948A>G)
BRCA1(c.2077G>A)
BRCA1(c.2612C>T)
BRCA1(c.3113A>G)
BRCA1(c.3548A>G)
BRCA1(c.4900A>G)
BRCA2(c.1114A>C)
BRCA2(c.7397T>C)
EGFR(c.1427G>A)
ERBB2(c.1963A>G)
FGFR1(c.1424C>T)
FLT3(c.680C>T)
IDH1(c.532G>A)
KRAS(c.35G>A)
PIK3CA(c.3140A>T)
RET(c.207G>A)
STK11(c.1062C>G)
TP53(c.215C>G)
PDOX
PDO
PDO_LP
PT
1
0.8
0.6
0.4
0.2
0
Copy ratio (log2)
VAF

Figure S2 Whole exome sequencing on PDMC models.

(A) Venn diagram showing most of the missense mutations were shared among PDMC models [patient (PT), PDO, PDOX, and PDO-later passage (PDO_LP)].

(B) Driver mutations were largely represented among PDMC models.

(C) The distribution patterns of CNV and VAF in PDMC.

Figure S3

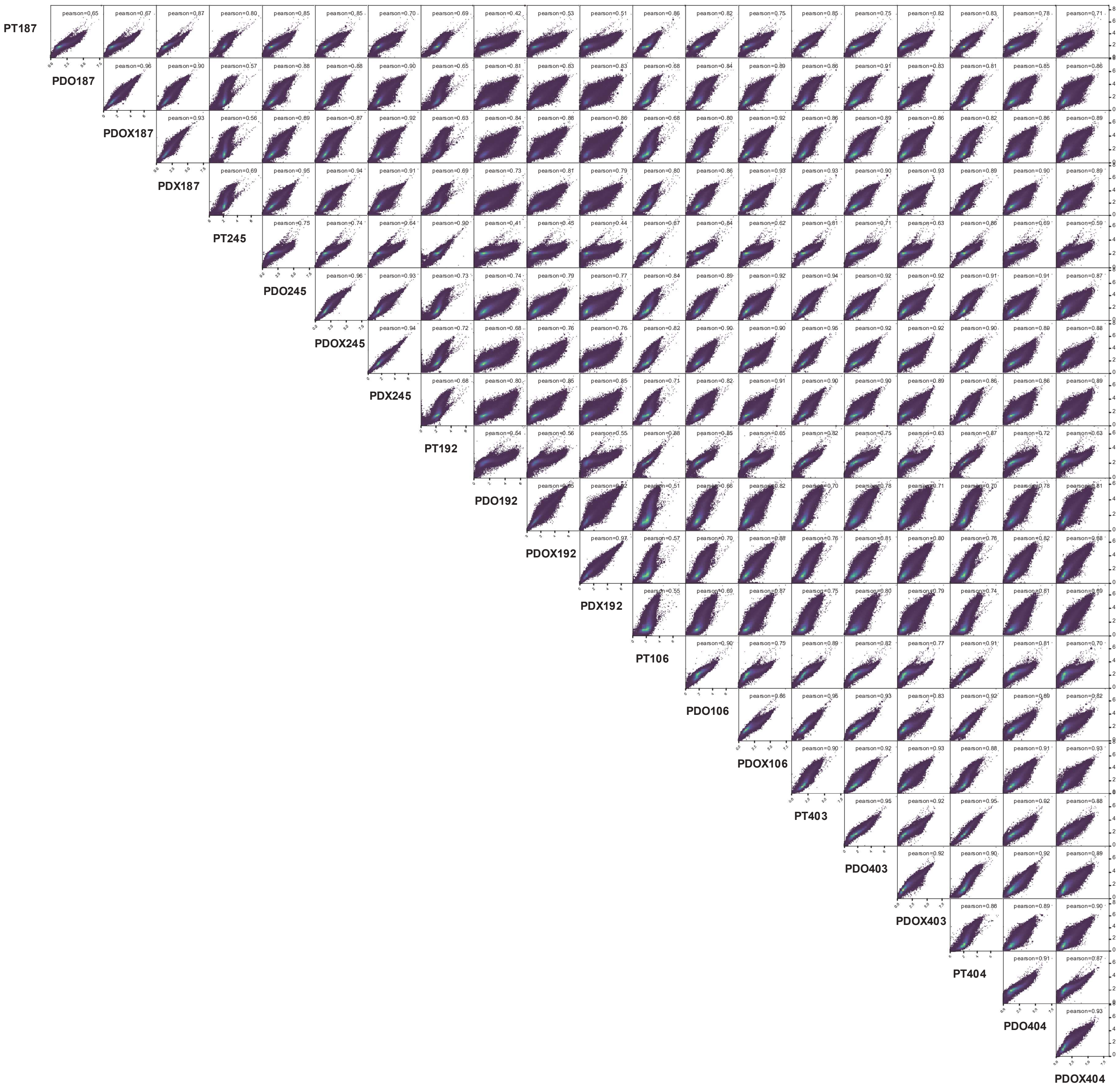
PT187
PDO187
PDOX187
PDX187
PT245
PDO245
PDOX245
PDX245
PT192
PDO192
PDOX192
PDX192
PT106
PDO106
PDOX106
PT403
PDO403
PDOX403
PT404
PDO404
PDOX404

Figure S3 Pairwise ATAC-seq correlation between different replicates of patient samples and models based on normalized read counts of detected peaks. Pearson correlation coefficient is presented for each comparison.

Figure S4

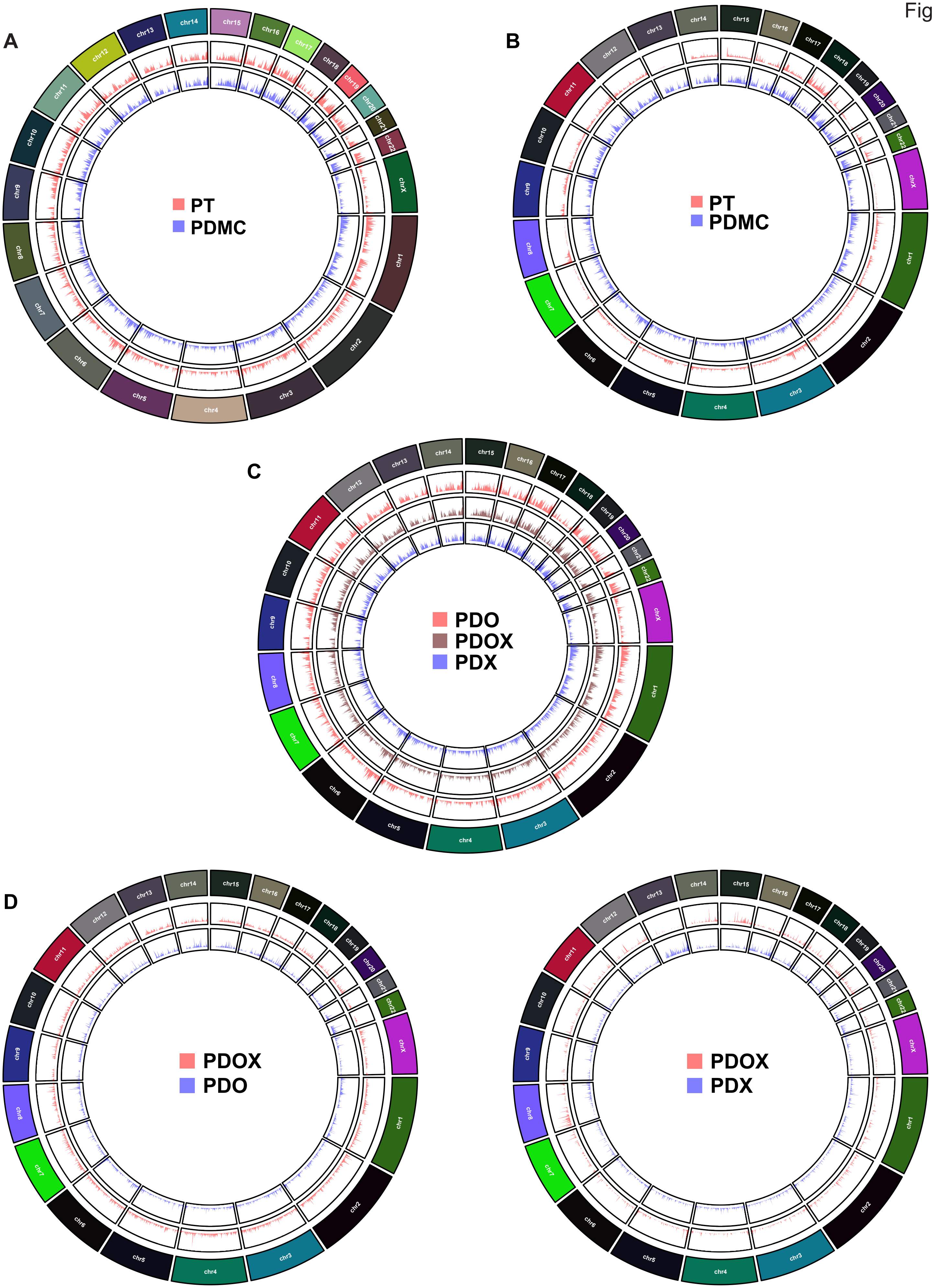

Figure S4 Circos plots based on ATAC-seq data.

(A) Circos plot showing the global chromatin accessibility based on ATAC-seq. The accumulated ATAC-seq peak density was presented in peak positions within the chromosomes. The peaks of PT are shown in red, and the peaks of PDMC are blue.

(B) Circos plot showing the DE peaks density of the comparison of PT vs. PDMC ($|log2(FC)|>1$, $p<0.05$).

(C) Circos plot showing the global chromatin accessibilities of PDO, PDOX, and PDX.

(D) Circos plots showing the DE peaks density of the comparison of PDOX vs. PDO and PDOX vs. PDX ($|log2(FC)|>1$, $p<0.05$).

Figure S5

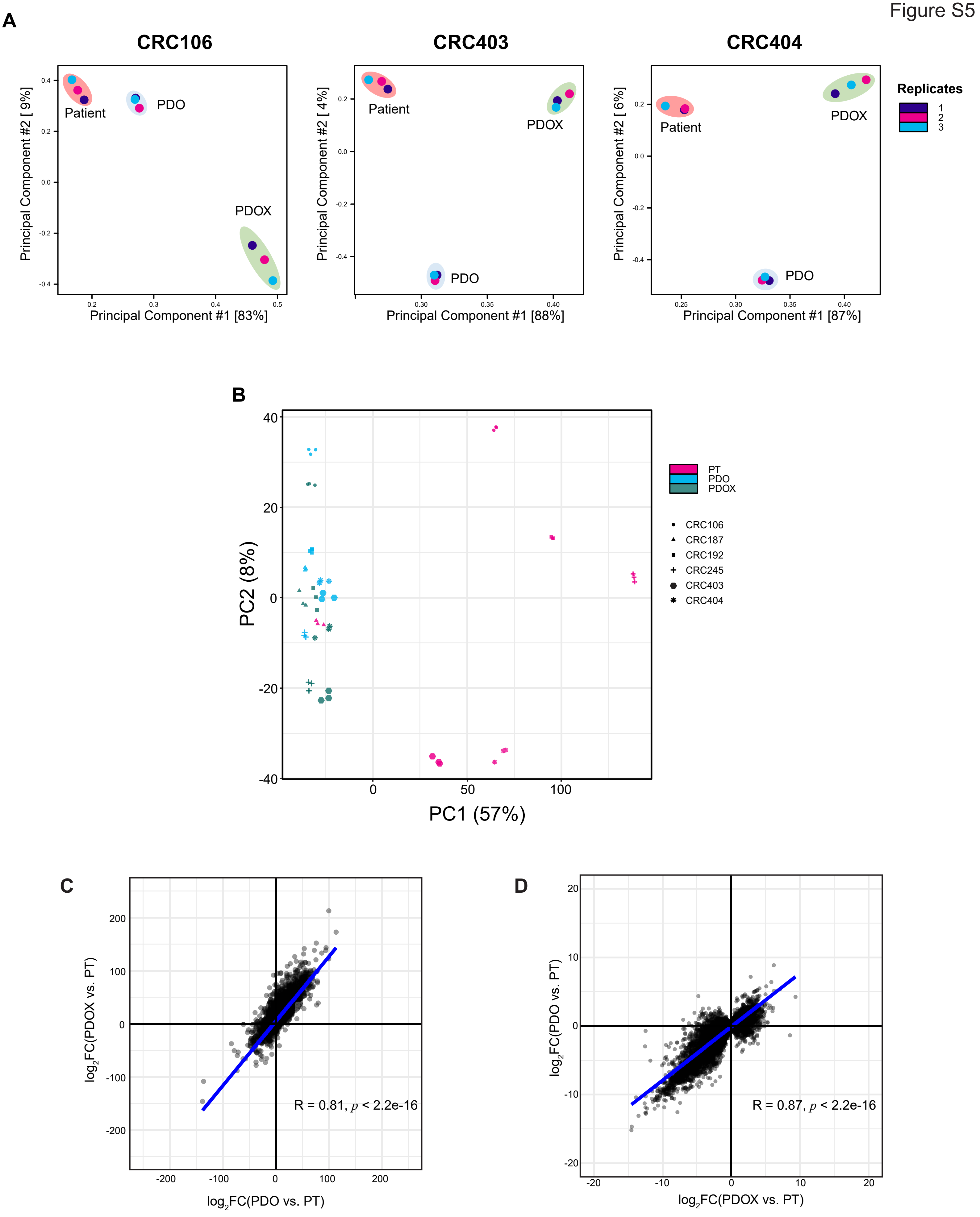

A
CRC106
CRC403
CRC404
Patient
PDO
PDOX
Principal Component #1 [83%]
Principal Component #2 [9%]
Principal Component #1 [88%]
Principal Component #2 [4%]
Principal Component #1 [87%]
Principal Component #2 [6%]
Replicates
1
2
3
B
PC1 (57%)
PC2 (8%)
PT
PDO
PDOX
CRC106
CRC187
CRC192
CRC245
CRC403
CRC404
C
log2FC(PDO vs. PT)
log2FC(PDOX vs. PT)
R = 0.81, p < 2.2e-16
D
log2FC(PDOX vs. PT)
log2FC(PDO vs. PT)
R = 0.87, p < 2.2e-16

Figure S5 Supplemental principal component analysis and correlation analysis.

(A) Principal component analysis of individual PT-PDMC sets (CRC106, CRC403, and CRC404) from ATAC-seq data based on the DiffBind score DBA_SCORE_RPKM. Patient samples are circled in red, PDO in blue, and PDOX in light green. Each type of model has three replicates.

(B) Principal component analysis of the pooled PT-PDO-PDOX sets based on mRNA-seq data. Magenta indicates patient samples, blue indicates PDO, and green indicates PDOX. Different shapes of dots denote patient IDs.

(C) Correlation analysis of PDOX vs. PT and PDO vs. PT based on the differential enrichment (log2FC) ATAC-seq. Pearson correlation R value is 0.81.

(D) Correlation analysis of PDOX vs. PT and PDO vs. PT based on the differential expression (log2FC) in mRNA-seq. Pearson correlation R value is 0.87.

Figure S6

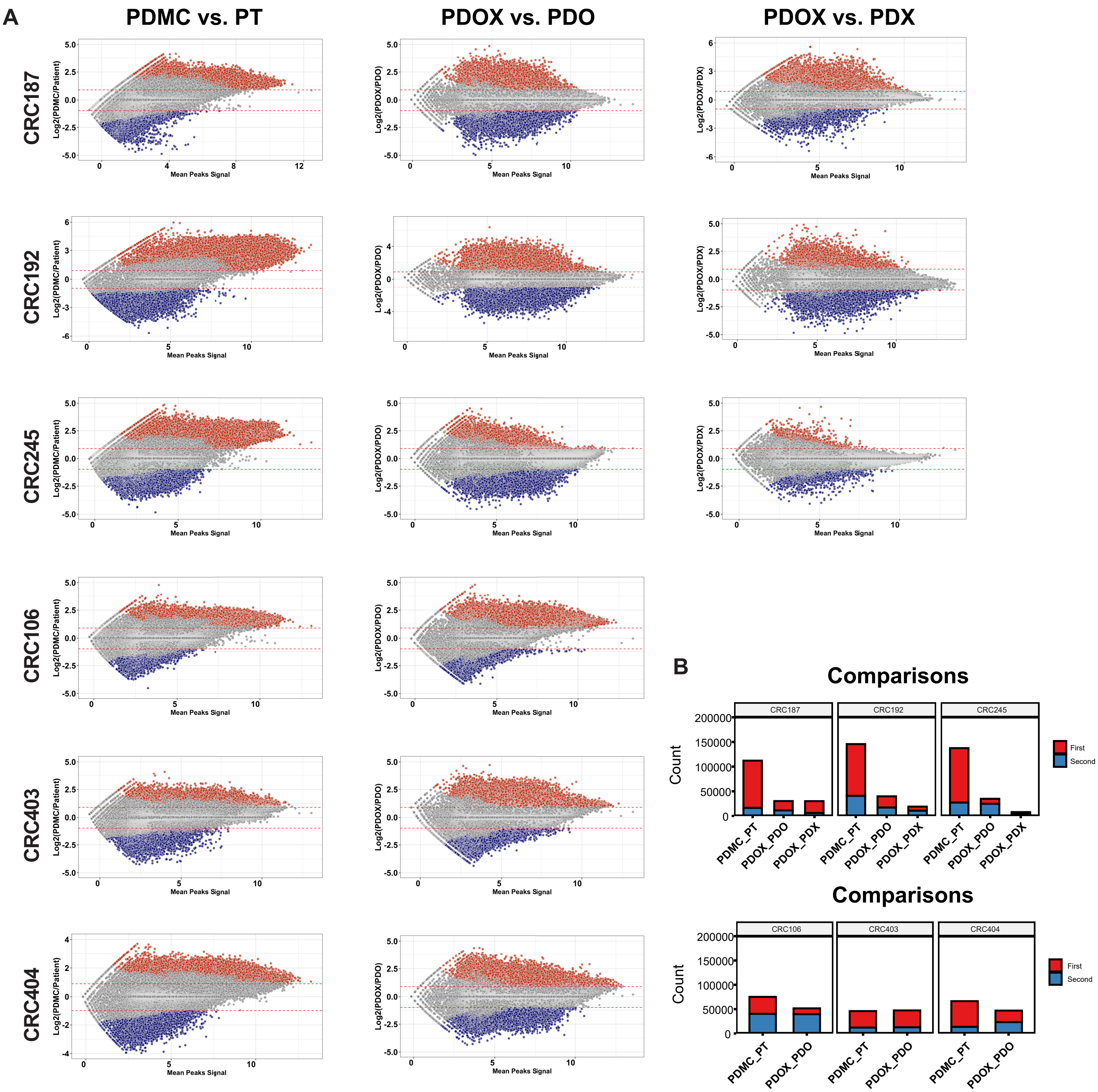

A
PDMC vs. PT
PDOX vs. PDO
PDOX vs. PDX
CRC187
CRC192
CRC245
CRC106
CRC403
CRC404
Log2(PDMC/Patient)
Log2(PDOX/PDO)
Log2(PDOX/PDX)
Mean Peaks Signal
B
Comparisons
CRC187
CRC192
CRC245
CRC106
CRC403
CRC404
Count
First
Second
PDMC_PT
PDOX_PDO
PDOX_PDX

Figure S6 Supplemental MA plots based on ATAC-seq data.

(A) ATAC-seq differential analysis of individual PT-PDMC sets. MA plots demonstrating the differential enriched peaks in each comparison ($|log2(FC)|>1$, $p<0.05$).

(B) The bar plots report the number of counts of the differentially enriched peaks.

Figure S7

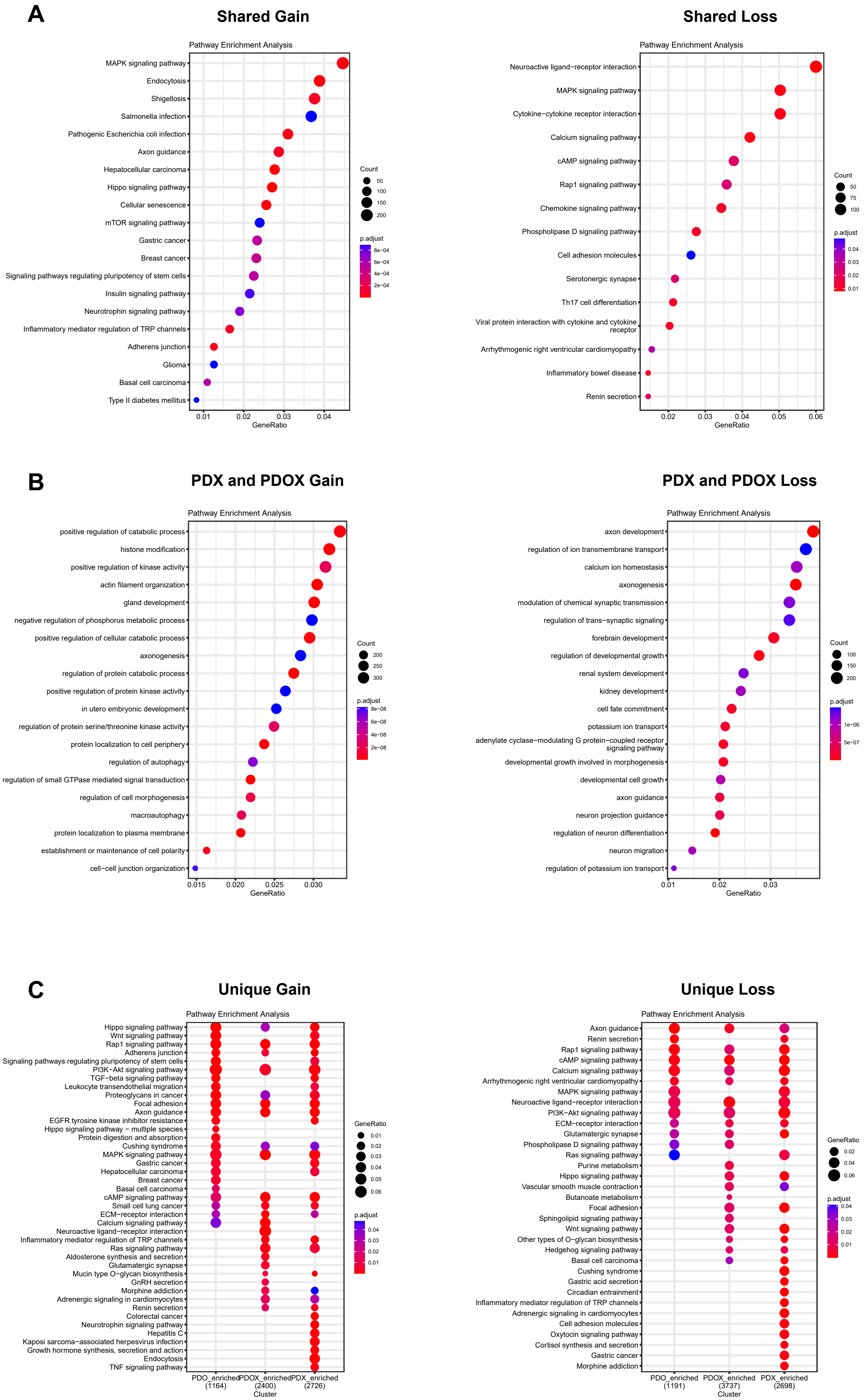
A
Shared Gain
Pathway Enrichment Analysis
MAPK signaling pathway
Endocytosis
Shigellosis
Salmonella infection
Pathogenic Escherichia coli infection
Axon guidance
Hepatocellular carcinoma
Hippo signaling pathway
Cellular senescence
mTOR signaling pathway
Gastric cancer
Breast cancer
Signaling pathways regulating pluripotency of stem cells
Insulin signaling pathway
Neurotrophin signaling pathway
Inflammatory mediator regulation of TRP channels
Adherens junction
Glioma
Basal cell carcinoma
Type II diabetes mellitus
0.01 0.02 0.03 0.04
GeneRatio
Count
50
100
150
200
p.adjust
8e−04
6e−04
4e−04
2e−04
Shared Loss
Pathway Enrichment Analysis
Neuroactive ligand−receptor interaction
MAPK signaling pathway
Cytokine−cytokine receptor interaction
Calcium signaling pathway
cAMP signaling pathway
Rap1 signaling pathway
Chemokine signaling pathway
Phospholipase D signaling pathway
Cell adhesion molecules
Serotonergic synapse
Th17 cell differentiation
Viral protein interaction with cytokine and cytokine receptor
Arrhythmogenic right ventricular cardiomyopathy
Inflammatory bowel disease
Renin secretion
0.02 0.03 0.04 0.05 0.06
GeneRatio
Count
50
75
100
p.adjust
0.04
0.03
0.02
0.01
B
PDX and PDOX Gain
Pathway Enrichment Analysis
positive regulation of catabolic process
histone modification
positive regulation of kinase activity
actin filament organization
gland development
negative regulation of phosphorus metabolic process
positive regulation of cellular catabolic process
axonogenesis
regulation of protein catabolic process
positive regulation of protein kinase activity
in utero embryonic development
regulation of protein serine/threonine kinase activity
protein localization to cell periphery
regulation of autophagy
regulation of small GTPase mediated signal transduction
regulation of cell morphogenesis
macroautophagy
protein localization to plasma membrane
establishment or maintenance of cell polarity
cell−cell junction organization
0.015 0.020 0.025 0.030
GeneRatio
Count
200
250
300
p.adjust
8e−08
6e−08
4e−08
2e−08
PDX and PDOX Loss
Pathway Enrichment Analysis
axon development
regulation of ion transmembrane transport
calcium ion homeostasis
axonogenesis
modulation of chemical synaptic transmission
regulation of trans−synaptic signaling
forebrain development
regulation of developmental growth
renal system development
kidney development
cell fate commitment
potassium ion transport
adenylate cyclase−modulating G protein−coupled receptor signaling pathway
developmental growth involved in morphogenesis
developmental cell growth
axon guidance
neuron projection guidance
regulation of neuron differentiation
neuron migration
regulation of potassium ion transport
0.01 0.02 0.03
GeneRatio
Count
100
150
200
p.adjust
1e−06
5e−07
C
Unique Gain
Pathway Enrichment Analysis
Hippo signaling pathway
Wnt signaling pathway
Rap1 signaling pathway
Adherens junction
Signaling pathways regulating pluripotency of stem cells
PI3K−Akt signaling pathway
TGF−beta signaling pathway
Leukocyte transendothelial migration
Proteoglycans in cancer
Focal adhesion
Axon guidance
EGFR tyrosine kinase inhibitor resistance
Hippo signaling pathway − multiple species
Protein digestion and absorption
Cushing syndrome
MAPK signaling pathway
Gastric cancer
Hepatocellular carcinoma
Breast cancer
Basal cell carcinoma
cAMP signaling pathway
Small cell lung cancer
ECM−receptor interaction
Calcium signaling pathway
Neuroactive ligand−receptor interaction
Inflammatory mediator regulation of TRP channels
Ras signaling pathway
Aldosterone synthesis and secretion
Glutamatergic synapse
Mucin type O−glycan biosynthesis
GnRH secretion
Morphine addiction
Adrenergic signaling in cardiomyocytes
Renin secretion
Colorectal cancer
Neurotrophin signaling pathway
Hepatitis C
Kaposi sarcoma−associated herpesvirus infection
Growth hormone synthesis, secretion and action
Endocytosis
TNF signaling pathway
PDO_enriched (1164)
PDOX_enriched (2400)
PDX_enriched (2726)
Cluster
GeneRatio
0.01
0.02
0.03
0.04
0.05
0.06
p.adjust
0.04
0.03
0.02
0.01
Unique Loss
Pathway Enrichment Analysis
Axon guidance
Renin secretion
Rap1 signaling pathway
cAMP signaling pathway
Calcium signaling pathway
Arrhythmogenic right ventricular cardiomyopathy
MAPK signaling pathway
Neuroactive ligand−receptor interaction
PI3K−Akt signaling pathway
ECM−receptor interaction
Glutamatergic synapse
Phospholipase D signaling pathway
Ras signaling pathway
Purine metabolism
Hippo signaling pathway
Vascular smooth muscle contraction
Butanoate metabolism
Focal adhesion
Sphingolipid signaling pathway
Wnt signaling pathway
Other types of O−glycan biosynthesis
Hedgehog signaling pathway
Basal cell carcinoma
Cushing syndrome
Gastric acid secretion
Circadian entrainment
Inflammatory mediator regulation of TRP channels
Adrenergic signaling in cardiomyocytes
Cell adhesion molecules
Oxytocin signaling pathway
Cortisol synthesis and secretion
Gastric cancer
Morphine addiction
PDO_enriched (1191)
PDOX_enriched (3737)
PDX_enriched (2698)
Cluster
GeneRatio
0.02
0.04
0.06
p.adjust
0.04
0.03
0.02
0.01

Figure S7 The pathway analysis.

(A) KEGG pathways enriched in the shared gain or loss peaks defined in Figure 2I.

(B) GeneOntology biological processes enriched in the shared gain/loss peaks in PDX and PDOX as defined in Figure 2I.

(C) KEGG pathways enriched in the unique gain/loss peaks for PDO, PDOX, and PDX as defined in Figure 2I.

Figure S8

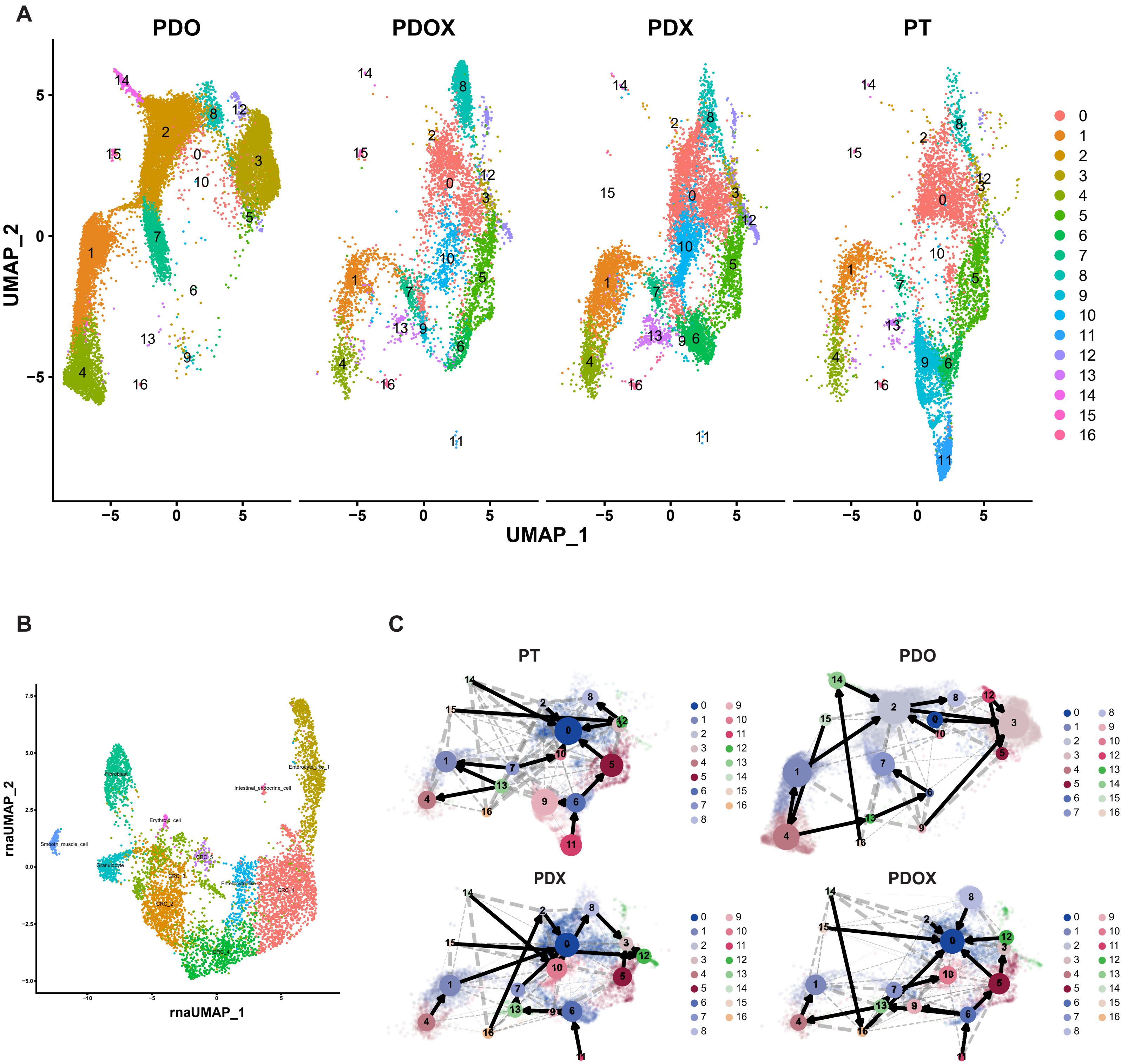
A
PDO
PDOX
PDX
PT
UMAP_2
UMAP_1
B
rnaUMAP_2
rnaUMAP_1
Smooth_muscle_cell
Granulocyte
Erythroid_cell
Intestinal_endocrine_cell
Enterocyte_like_1
C
PT
PDO
PDX
PDOX

Figure S8 Supplemental single cell multiome analysis.

(A) Integrated UMAP of PDO, PDOX, PDX, and PT samples from the CRC187 set, clustering based on gene expressions.

(B) Visualizations of the cell clusters based on the gene expression in the PT sample only, showing the cell types of non-malignant or CRC components.

(C) RNA velocity analysis based on 10X single cell multiomic data.

Figure S9

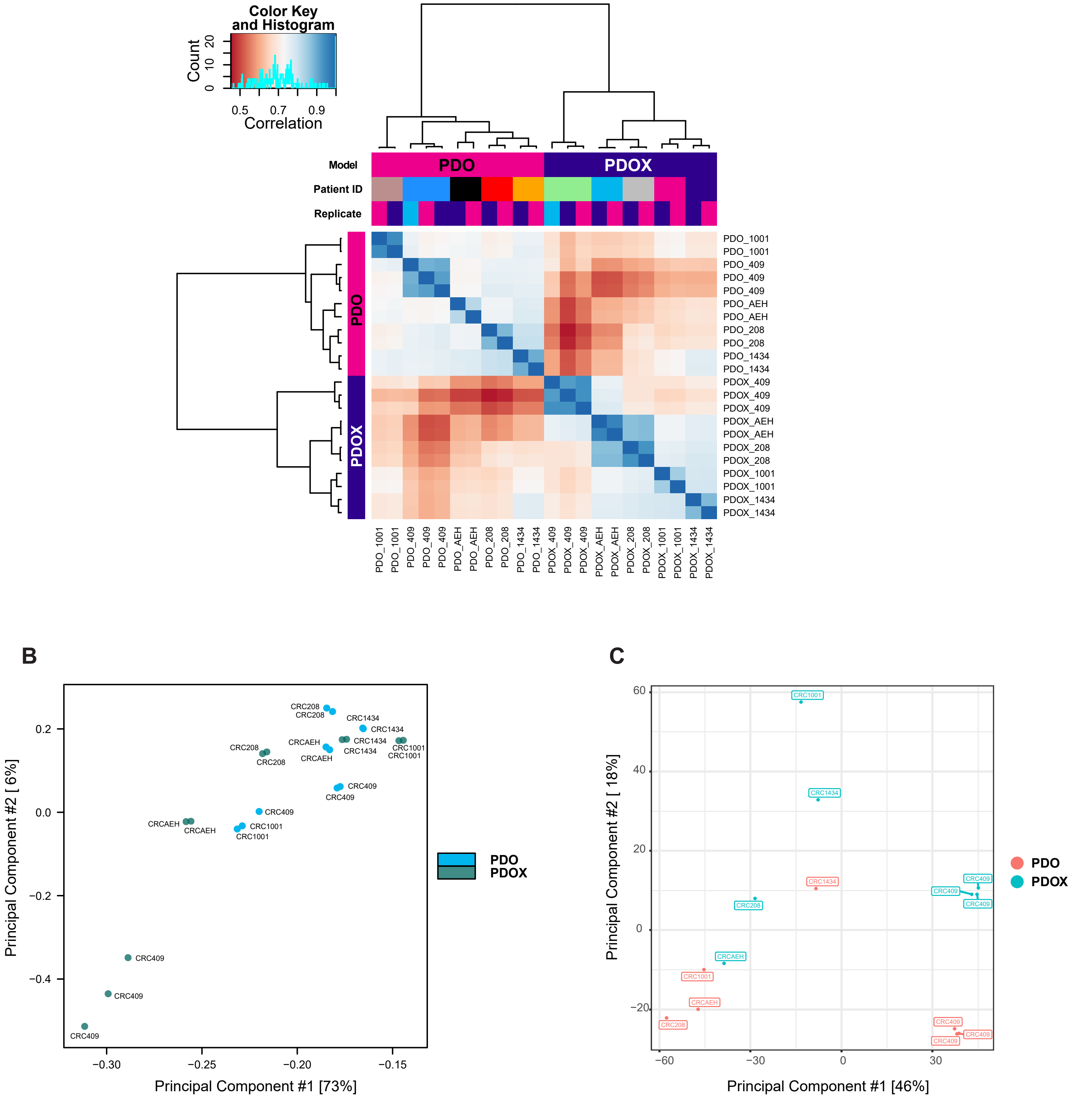
A
Color Key and Histogram
Count
0 10 20
0.5 0.7 0.9
Correlation
Model
Patient ID
Replicate
PDO
PDOX
PDO_1001
PDO_1001
PDO_409
PDO_409
PDO_409
PDO_AEH
PDO_AEH
PDO_208
PDO_208
PDO_1434
PDO_1434
PDOX_409
PDOX_409
PDOX_409
PDOX_AEH
PDOX_AEH
PDOX_208
PDOX_208
PDOX_1001
PDOX_1001
PDOX_1434
PDOX_1434
B
Principal Component #1 [73%]
Principal Component #2 [6%]
−0.30 −0.25 −0.20 −0.15
0.2 0.0 −0.2 −0.4
CRC208
CRC1434
CRCAEH
CRC1001
CRC409
PDO
PDOX
C
Principal Component #1 [46%]
Principal Component #2 [18%]
−60 −30 0 30
60 40 20 0 −20
CRC1001
CRC1434
CRC208
CRCAEH
CRC409
PDO
PDOX

Figure S9 Analysis of the additional PDO-PDOX sets.

(A) Heatmap of unbiased clustering of five additional PDO-PDOX sets based on ATAC-seq, showing separation of PDOX and PDO.

(B) Principal component analysis of the additional PDO-PDOX sets based on ATAC-seq data.

(C) Principal component analysis of the additional PDO-PDOX sets based on mRNA-seq data.

Figure S10

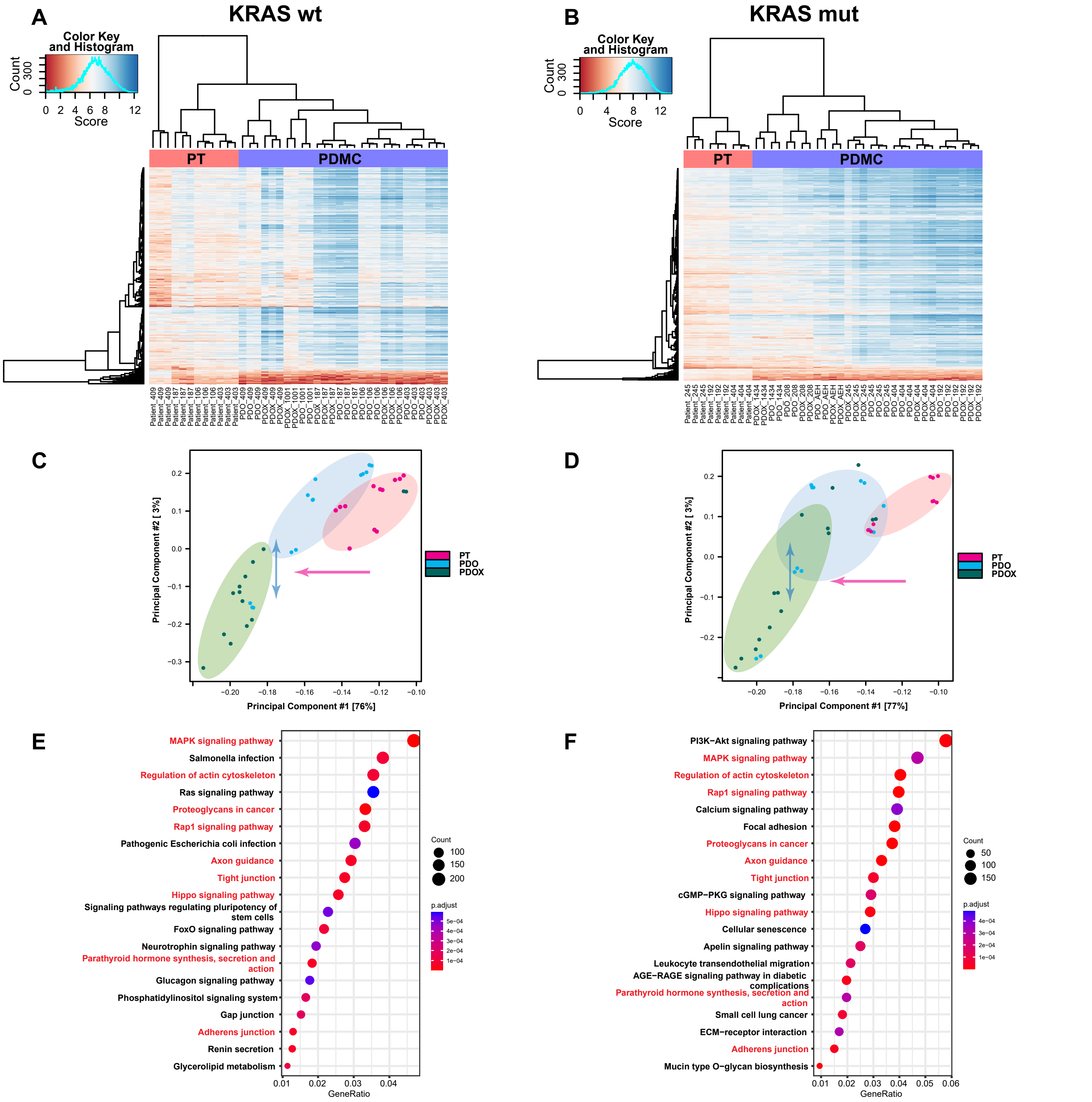

A
KRAS wt
Color Key
and Histogram
Count
Score
PT
PDMC
B
KRAS mut
Color Key
and Histogram
Count
Score
PT
PDMC
C
Principal Component #1 [76%]
Principal Component #2 [3%]
PT
PDO
PDOX
D
Principal Component #1 [77%]
Principal Component #2 [3%]
PT
PDO
PDOX
E
MAPK signaling pathway
Salmonella infection
Regulation of actin cytoskeleton
Ras signaling pathway
Proteoglycans in cancer
Rap1 signaling pathway
Pathogenic Escherichia coli infection
Axon guidance
Tight junction
Hippo signaling pathway
Signaling pathways regulating pluripotency of stem cells
FoxO signaling pathway
Neurotrophin signaling pathway
Parathyroid hormone synthesis, secretion and action
Glucagon signaling pathway
Phosphatidylinositol signaling system
Gap junction
Adherens junction
Renin secretion
Glycerolipid metabolism
GeneRatio
Count
p.adjust
F
PI3K−Akt signaling pathway
MAPK signaling pathway
Regulation of actin cytoskeleton
Rap1 signaling pathway
Calcium signaling pathway
Focal adhesion
Proteoglycans in cancer
Axon guidance
Tight junction
cGMP−PKG signaling pathway
Hippo signaling pathway
Cellular senescence
Apelin signaling pathway
Leukocyte transendothelial migration
AGE−RAGE signaling pathway in diabetic complications
Parathyroid hormone synthesis, secretion and action
Small cell lung cancer
ECM−receptor interaction
Adherens junction
Mucin type O−glycan biosynthesis
GeneRatio
Count
p.adjust

Figure S10 ATAC-seq analysis on different genomic sub-types and passages.

(A-B) Heatmaps of non-biased hierarchical clustering of patient tissue (PT) and PDMC of wild-type **(A)** and mutant **(B)** KRAS groups based on ATAC-seq results.

(C-D) Principal component analysis of wild-type **(C)** and mutant **(D)** KRAS groups based on ATAC-seq results. Patient samples are in magenta, PDO are in blue, and PDOX are in green.

(E-F) PDOX enriched KEGG pathways in the comparison of PDOX vs. PDO from wild-type **(E)** and mutant **(F)** KRAS groups based on the differentially enriched ATAC-seq peaks. The shared pathways are labeled in red.

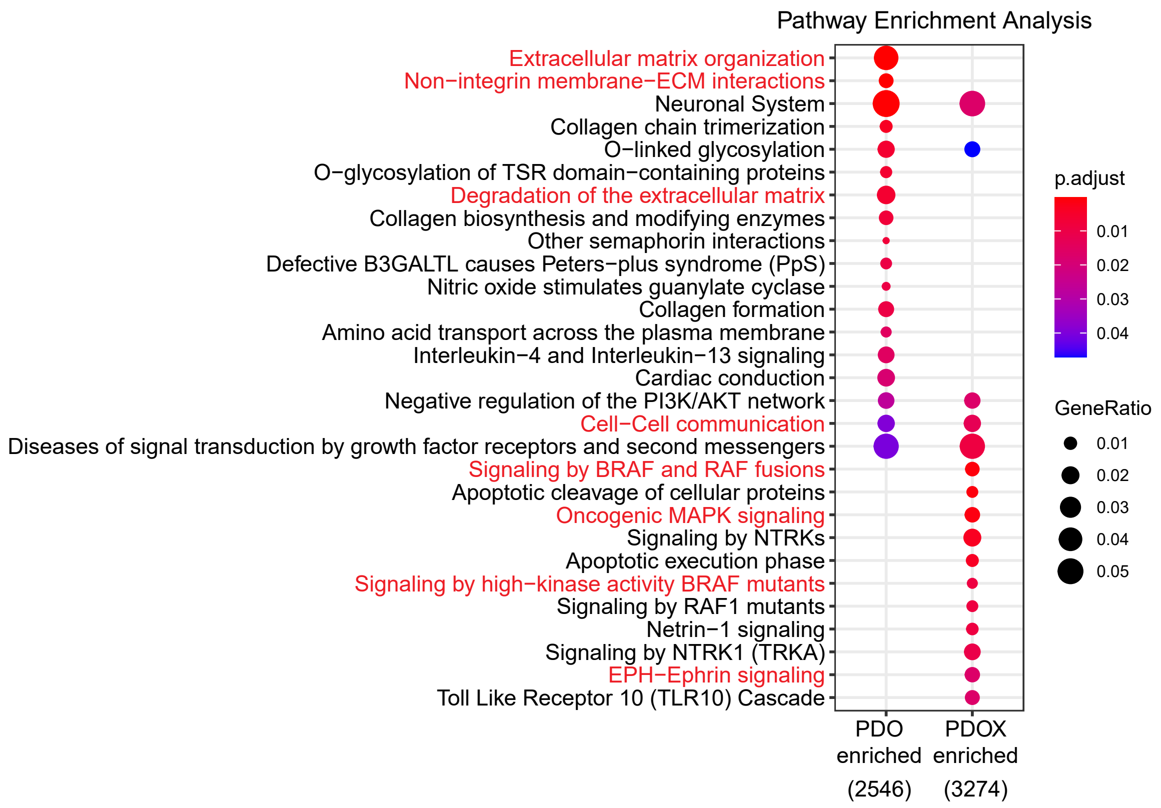
Pathway Enrichment Analysis
Extracellular matrix organization
Non−integrin membrane−ECM interactions
Neuronal System
Collagen chain trimerization
O−linked glycosylation
O−glycosylation of TSR domain−containing proteins
Degradation of the extracellular matrix
Collagen biosynthesis and modifying enzymes
Other semaphorin interactions
Defective B3GALTL causes Peters−plus syndrome (PpS)
Nitric oxide stimulates guanylate cyclase
Collagen formation
Amino acid transport across the plasma membrane
Interleukin−4 and Interleukin−13 signaling
Cardiac conduction
Negative regulation of the PI3K/AKT network
Cell−Cell communication
Diseases of signal transduction by growth factor receptors and second messengers
Signaling by BRAF and RAF fusions
Apoptotic cleavage of cellular proteins
Oncogenic MAPK signaling
Signaling by NTRKs
Apoptotic execution phase
Signaling by high−kinase activity BRAF mutants
Signaling by RAF1 mutants
Netrin−1 signaling
Signaling by NTRK1 (TRKA)
EPH−Ephrin signaling
Toll Like Receptor 10 (TLR10) Cascade
PDO enriched (2546)
PDOX enriched (3274)
p.adjust
0.01
0.02
0.03
0.04
GeneRatio
0.01
0.02
0.03
0.04
0.05

Figure S11 Pathway Enrichment Analysis of PDOX vs. PDO.

KEGG pathways enrichment analysis based on the differentially enriched peaks in the comparison of PDOX vs. PDO.

Figure S12

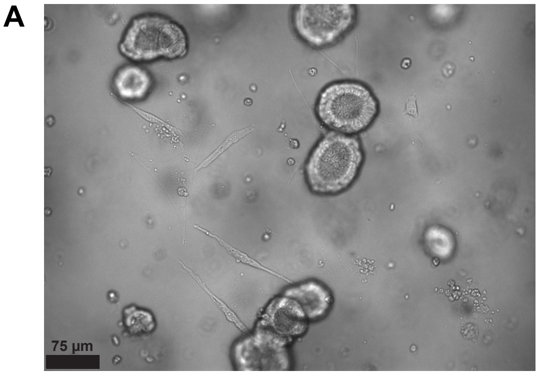
A
75 µm

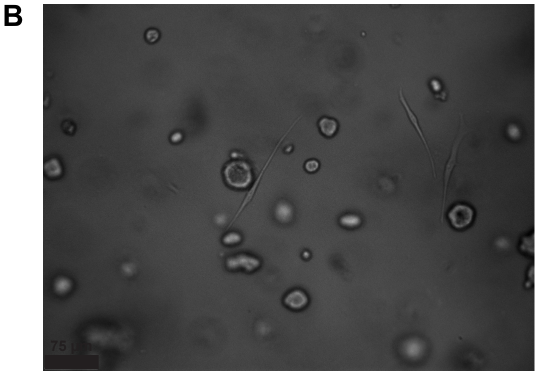
B

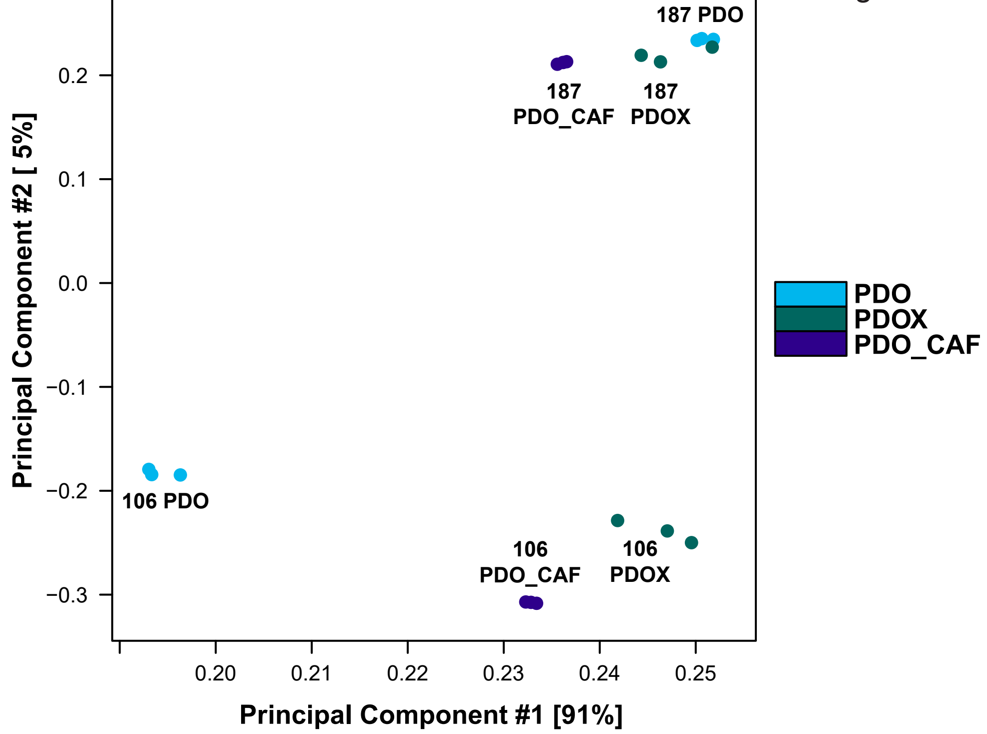
187 PDO
187
PDO_CAF
187
PDOX
106 PDO
106
PDO_CAF
106
PDOX
Principal Component #2 [5%]
Principal Component #1 [91%]
0.2
0.1
0.0
−0.1
−0.2
−0.3
0.20
0.21
0.22
0.23
0.24
0.25
PDO
PDOX
PDO_CAF

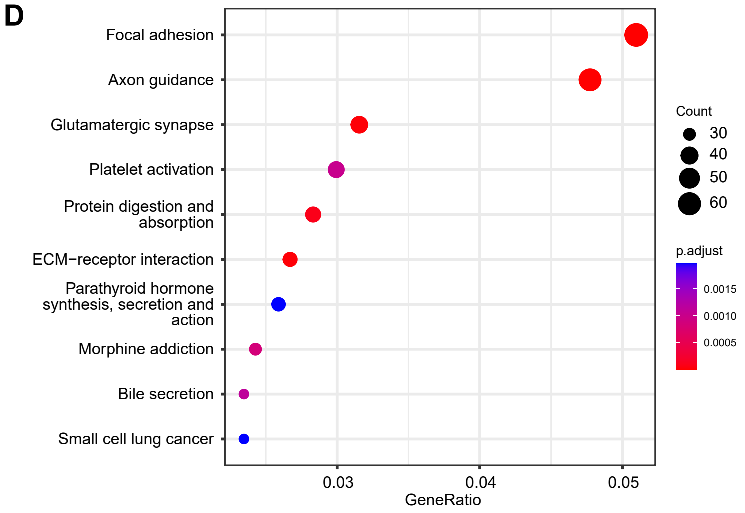
D
Focal adhesion
Axon guidance
Glutamatergic synapse
Platelet activation
Protein digestion and absorption
ECM−receptor interaction
Parathyroid hormone synthesis, secretion and action
Morphine addiction
Bile secretion
Small cell lung cancer
0.03
0.04
0.05
GeneRatio
Count
30
40
50
60
p.adjust
0.0015
0.0010
0.0005

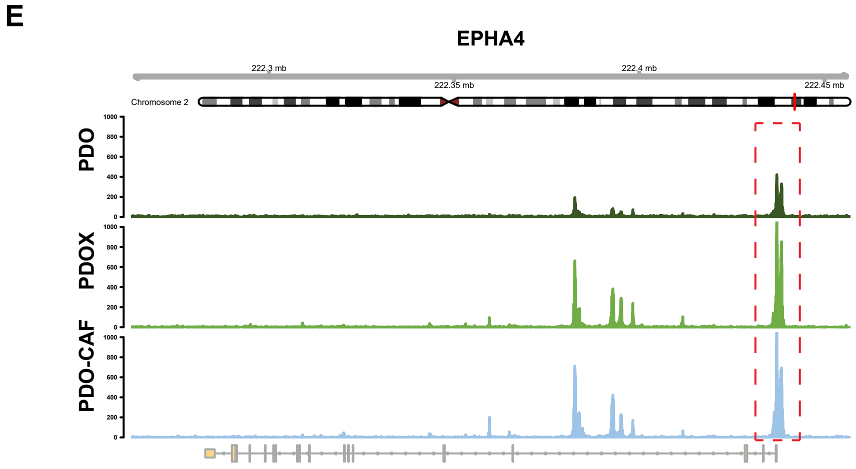
E
EPHA4
222.3 mb
222.35 mb
222.4 mb
222.45 mb
Chromosome 2
PDO
PDOX
PDO-CAF

Figure S12 The coculture experiment of PDO and matched cancer-associated fibroblasts (CAF).

(A-B) The bright-field images of PDO187 **(A)** and PDO106 **(B)** cocultured with their matched CAF.

(C) Principal component analysis showing PDO_CAF is more similar to PDOX than PDO.

(D) KEGG pathways enrichment analysis based on the differentially enriched ATAC peaks in the PDO_CAF.

(E) ATAC-seq signal tracks showing EPHA4 locus in PDO, PDOX, and PDO-CAF models. The promoter regions are boxed.

Figure S13

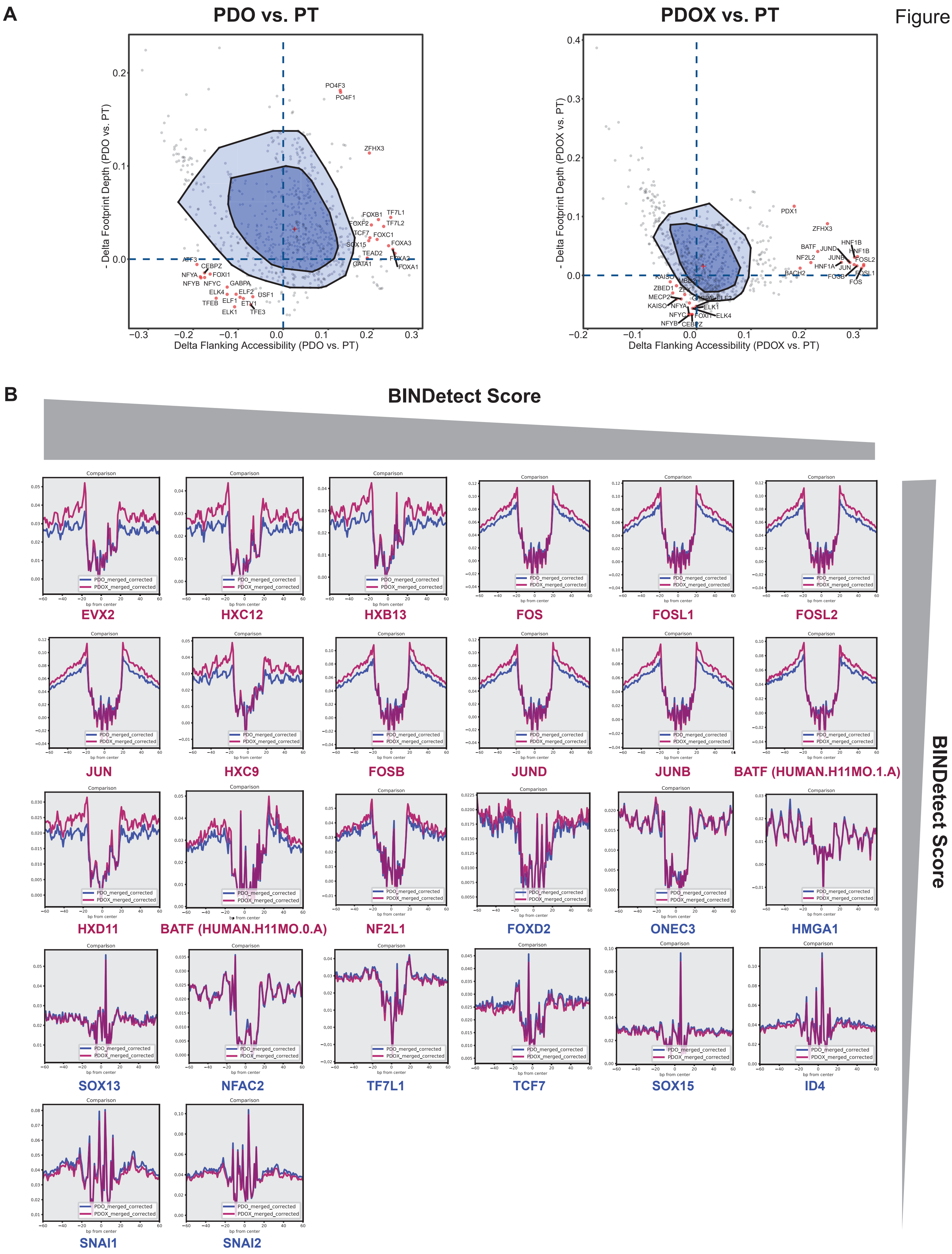

A
PDO vs. PT
Delta Flanking Accessibility (PDO vs. PT)
- Delta Footprint Depth (PDO vs. PT)
PDOX vs. PT
Delta Flanking Accessibility (PDOX vs. PT)
- Delta Footprint Depth (PDOX vs. PT)
B
BINDetect Score
Comparison
bp from center
PDO_merged_corrected
PDOX_merged_corrected
EVX2
HXC12
HXB13
FOS
FOSL1
FOSL2
JUN
HXC9
FOSB
JUND
JUNB
BATF (HUMAN.H11MO.1.A)
HXD11
BATF (HUMAN.H11MO.0.A)
NF2L1
FOXD2
ONEC3
HMGA1
SOX13
NFAC2
TF7L1
TCF7
SOX15
ID4
SNAI1
SNAI2
BINDetect Score

Figure S13 Supplemental footprint analyses.

(A) BaGFoot analysis of PDO vs. PT and PDOX vs. PT. The TFs predicted to be active in PDO or PDOX are in the first quadrant beyond the fence area. The TFs predicted to be active in PT are in the third quadrant beyond the fence area. The top 15 TFs in each quadrant based on the distance to the origin are shown.

(B) Descending order of BINDetect Score showing the footprint analysis of the PDOX-active TFs (predicted to be beyond the fence in both comparisons of PDOX vs. PDO and PDOX vs. PT, with the TF names shown in red) and PDO-active TFs (predicted to be beyond the fence in both PDOX vs. PDO and PDO vs. PT, with the TF names shown in blue). For each TF, its footprints in PDOX and PDO are shown. The TFs are ranked by the BINDetect score (EVX2 has the highest absolute value of the active scores in PDOX, and SNAI2 has the highest absolute value of the active scores in PDO).

Figure S14

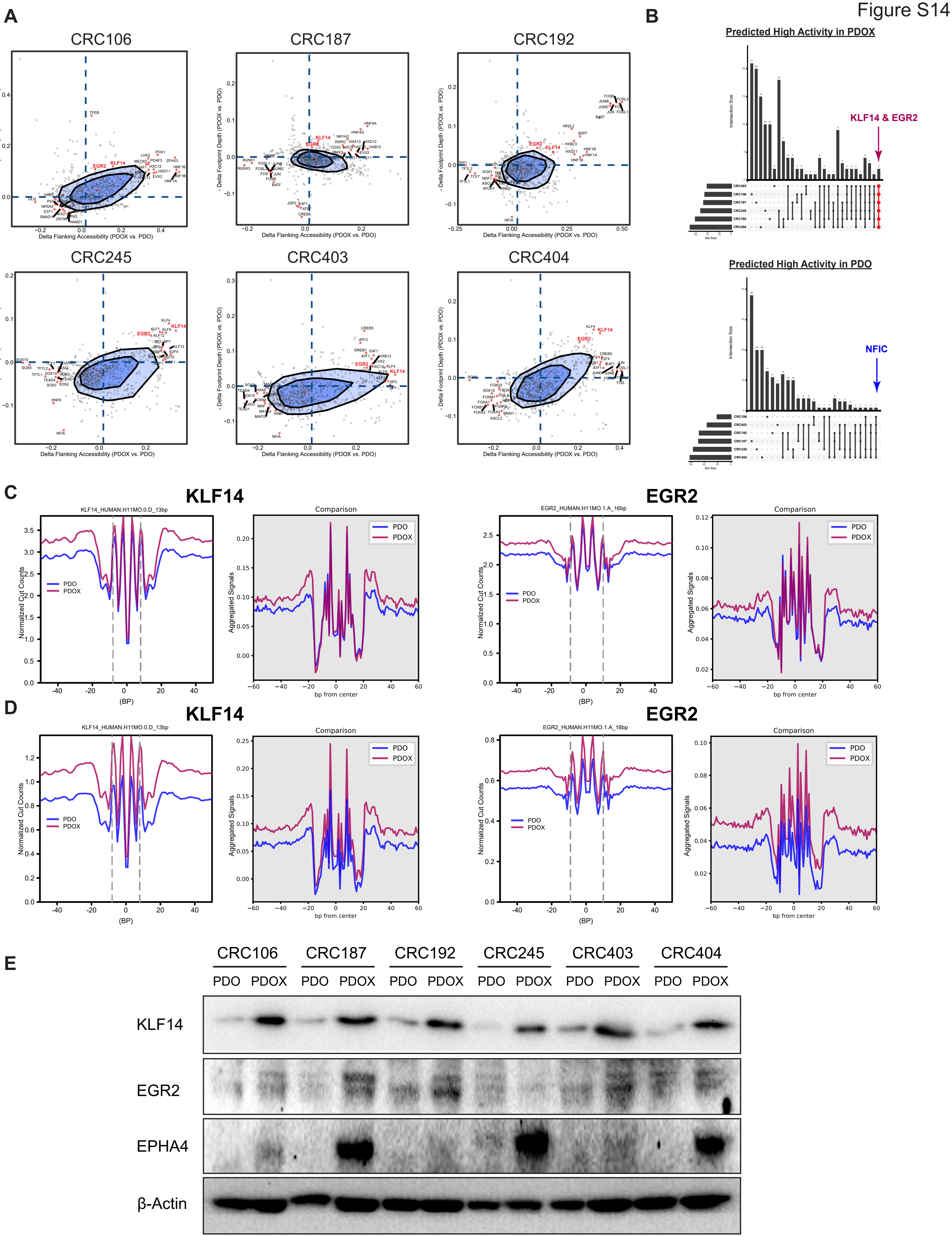

A
CRC106
CRC187
CRC192
CRC245
CRC403
CRC404
- Delta Footprint Depth (PDOX vs. PDO)
Delta Flanking Accessibility (PDOX vs. PDO)
KLF14
EGR2
B
Predicted High Activity in PDOX
KLF14 & EGR2
Predicted High Activity in PDO
NFIC
Intersection Size
Set Size
C
KLF14
KLF14_HUMAN.H11MO.0.D_13bp
Comparison
EGR2
EGR2_HUMAN.H11MO.1.A_16bp
Normalized Cut Counts
Aggregated Signals
(BP)
bp from center
PDO
PDOX
D
KLF14
EGR2
E
CRC106
CRC187
CRC192
CRC245
CRC403
CRC404
PDO
PDOX
KLF14
EGR2
EPHA4
β-Actin

Figure S14 BaGFoot analysis for individual patient sets.

(A) BaGFoot analysis of PDOX vs. PDO (by individual patient set). The top 15 TFs in the first and third quadrants based on the distance to the origin are shown.

(B) The upset plots summarizing the overlapped TFs among each individual patient set. KLF14 and EGR2 are predicted to be active in PDOX. NFIC is predicted to be active in PDO.

(C) KLF14 and EGR2 footprint comparisons of PDOX and PDO generated by BaGFoot (left for each panel) and BINDetect (right for each panel).

(D) Comparison of KLF14 and EGR2 footprints in the additional PDO-PDOX validation sets by BaGFoot (left for each panel) and BINDetect (right for each panel)

(E) Western blots showing the protein levels of KLF14, EGR2, and EPHA4 in matched PDO and PDOX.

Figure S15

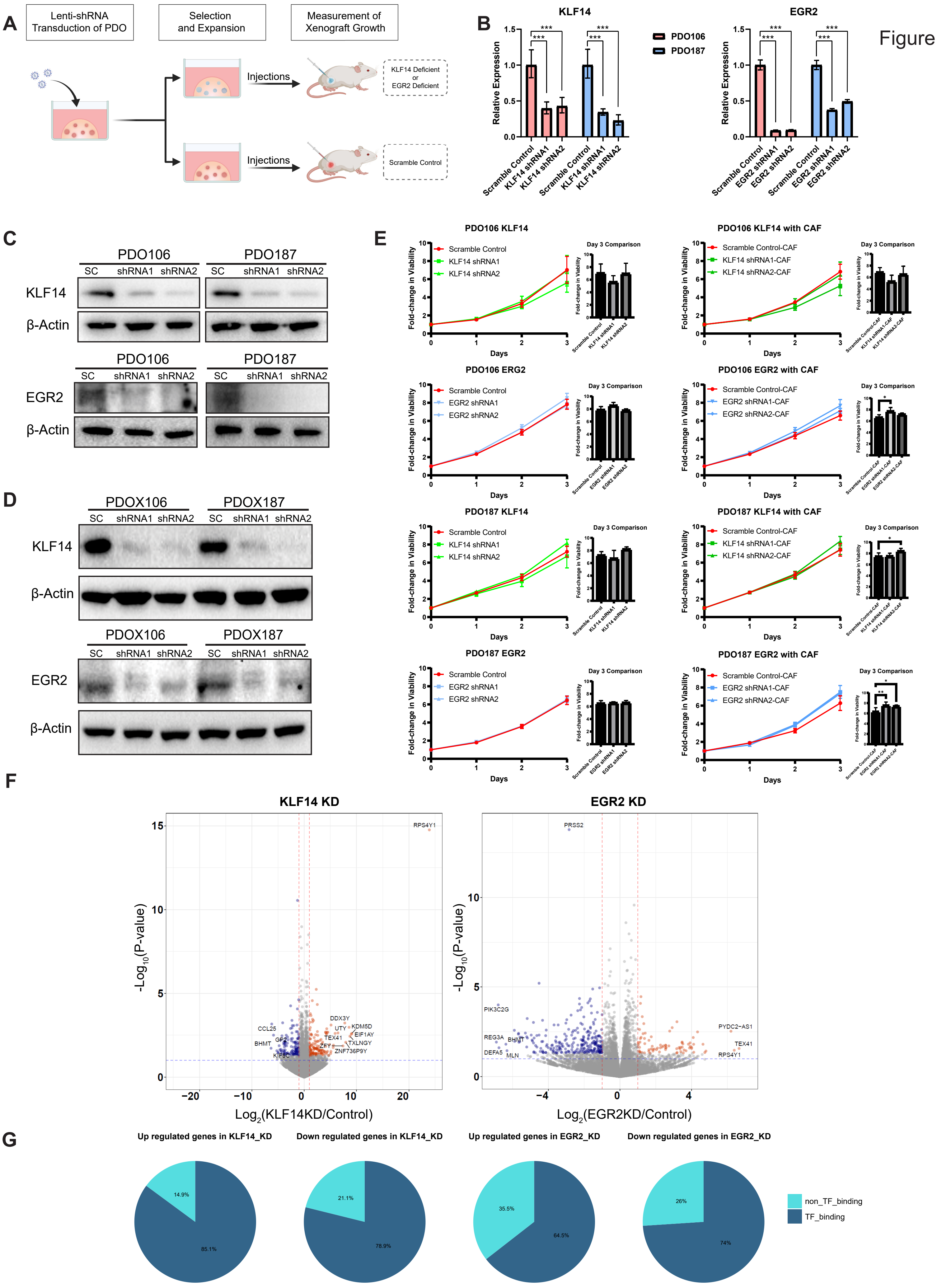
A
Lenti-shRNA Transduction of PDO
Selection and Expansion
Measurement of Xenograft Growth
Injections
Injections
KLF14 Deficient or EGR2 Deficient
Scramble Control
B
KLF14
EGR2
PDO106
PDO187
Relative Expression
Scramble Control
KLF14 shRNA1
KLF14 shRNA2
EGR2 shRNA1
EGR2 shRNA2
C
PDO106
PDO187
SC shRNA1 shRNA2
KLF14
β-Actin
EGR2
D
PDOX106
PDOX187
E
PDO106 KLF14
PDO106 KLF14 with CAF
PDO106 ERG2
PDO106 EGR2 with CAF
PDO187 KLF14
PDO187 KLF14 with CAF
PDO187 EGR2
PDO187 EGR2 with CAF
Day 3 Comparison
Fold-change in Viability
Days
Scramble Control-CAF
KLF14 shRNA1-CAF
KLF14 shRNA2-CAF
EGR2 shRNA1-CAF
EGR2 shRNA2-CAF
F
KLF14 KD
EGR2 KD
-Log10(P-value)
Log2(KLF14KD/Control)
Log2(EGR2KD/Control)
RPS4Y1
DDX3Y
UTY
KDM5D
EIF1AY
TEX41
TXLNGY
ZFY
ZNF736P9Y
CCL25
BHMT
KIF5C
PRSS2
PIK3C2G
REG3A
DEFA5
MLN
PYDC2−AS1
G
Up regulated genes in KLF14_KD
Down regulated genes in KLF14_KD
Up regulated genes in EGR2_KD
Down regulated genes in EGR2_KD
14.9%
85.1%
21.1%
78.9%
35.5%
64.5%
26%
74%
non_TF_binding
TF_binding

Figure S15 Supplemental KLF14 and EGR2 *in vivo* validations.

(A) Design of the *in vivo* validation using lenti-shRNA.

(B) qRT-PCR validated knocking down of KLF14 and EGR2 using the shRNAs. Error bars [relative quantification (RQ) minimum and maximum] indicate 95% confidence interval estimating the mean expressions (N=4). p values were calculated based on ANOVA using ΔCt, ***$p < 0.001$.

(C) Western blots showing knocking down of KLF14 and EGR2 in PDO using the shRNAs.

(D) Western blots showing knocking down of KLF14 and EGR2 in PDOX using the shRNAs.

(E) The growth of PDO without (Left) and with (Right) CAFs coculturing. The EPCAM positive areas (indicated by Alexa Fluor® 647) served as markers for CRC growth. All measurements were normalized to the initial Day 0 readings. *$p < 0.05$, **$p < 0.01$.

(F) Volcano plots showing differentially expressed genes (p-value <= 0.05 and |log2FC| >= 1) in PDOX upon KLF14 or EGR2 silencing.

(G) TF binding analysis in the RNA-seq differentially expressed genes upon KLF14/EGR2 silencing. Differentially expressed genes were overlapped with HOMER2 predicted binding sites (score > 8). The percentage of overlapping genes and non-overlapping genes were plotted by the pie charts.

Figure S16

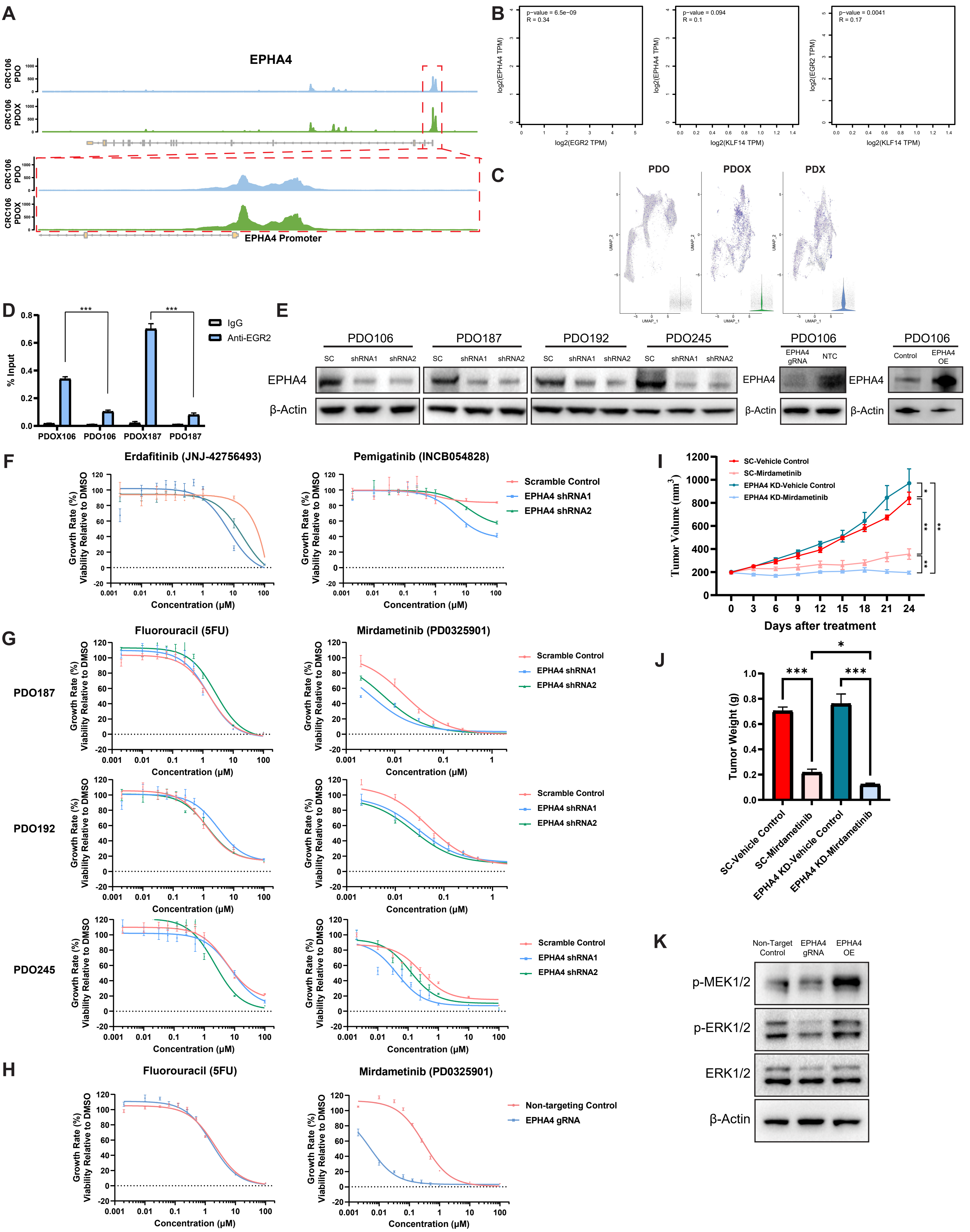
A
EPHA4
CRC106 PDO
CRC106 PDOX
EPHA4 Promoter
B
p-value = 6.5e−09
R = 0.34
p-value = 0.094
R = 0.1
p-value = 0.0041
R = 0.17
log2(EPHA4 TPM)
log2(EGR2 TPM)
log2(KLF14 TPM)
C
PDO
PDOX
PDX
D
% Input
IgG
Anti-EGR2
PDOX106
PDO106
PDOX187
PDO187
E
PDO106
PDO187
PDO192
PDO245
SC
shRNA1
shRNA2
EPHA4 gRNA
NTC
Control
EPHA4 OE
EPHA4
β-Actin
F
Erdafitinib (JNJ-42756493)
Pemigatinib (INCB054828)
Scramble Control
EPHA4 shRNA1
EPHA4 shRNA2
Growth Rate (%) Viability Relative to DMSO
Concentration (μM)
G
Fluorouracil (5FU)
Mirdametinib (PD0325901)
PDO187
PDO192
PDO245
H
Non-targeting Control
EPHA4 gRNA
I
SC-Vehicle Control
SC-Mirdametinib
EPHA4 KD-Vehicle Control
EPHA4 KD-Mirdametinib
Tumor Volume (mm3)
Days after treatment
J
Tumor Weight (g)
K
Non-Target Control
EPHA4 gRNA
EPHA4 OE
p-MEK1/2
p-ERK1/2
ERK1/2
β-Actin

Figure S16 EPHA4-related drug sensitivity tests.

(A) CRC106 ATAC-seq signal track showing EPHA4 locus in PDO and PDOX. The exon locations are indicated in the gene map. The promoter areas of EPHA4 are circled and presented in the bottom panel.

(B) Pearson correlation plots between EGR2 & EPHA4, KLF14 & EPHA4, and KLF14 & EGR2, based on expression data in TCGA.

(C) Visualizations of EPHA4 expression based on the single cell data. Insertions are violin plots summarizing the expressions of EPHA4 in different PDMC.

(D) ChIP-qPCR of anti-EGR2 on the promoter region of EPHA4. Percentage inputs for anti-EGR2 and IgG (negative control) were reported. Error bars denote SEM of three replicates. The p values were calculated based Student's t-test, ***$p < 0.001$.

(E) Western blots showing EPHA4 knocking down using the shRNAs (SC: Scramble control), EPHA4 knockout using gRNA (NTC: Non-targeting control), and overexpression (OE).

(F) PDO106 growth rate dose-response curves to Erdafitinib and Pemigatinib after knocking down EPHA4. Error bars denote SEM of four replicates.

(G) Growth rate dose-response curves of other PDO to Fluorouracil and Mirdametinib after knocking down EPHA4. Error bars denote SEM of four replicates.

(H) PDO106 growth rate dose-response curves to Fluorouracil and Mirdametinib after knocking out EPHA4. Error bars denote SEM of four replicates.

(I) Tumor growth curves of Scramble Control (SC) and EPHA4 KD PDOX106 treated with Mirdametinib (20 mg/kg/day for 24 days, oral gavage) or vehicle control. The treatments started when tumors reached a volume of 200 mm^3. Error bars denote SEM of five replicates. p values were calculated based on repeat measurement one-way ANOVA with Fisher's LSD test. *$p<0.05$, **$p<0.01$.

(J) Tumor weight comparisons of scramble control and EPHA4 KD PDOX106 treated with Mirdametinib or vehicle control. Error bars denote SEM of five replicates. p values were calculated based on ANOVA with post-hoc. *$p<0.05$, **$p < 0.01$, ***$p < 0.001$

(K) Western blots showing the expression levels of MEK/ERK when knocking-out or overexpressing (OE) EPHA4 in PDO.

Supplemental Tables

Supplemental Table 1 Summary of patient information

Patient ID	Sex	Age	Histology	Primary or Metastatic	Primary Tumor location	MSS or MSI
CRC106	Male	85	Adenocarcinoma	Primary CRC	Cecum	MSS
CRC187	Female	74	Adenocarcinoma	Primary CRC	Hepatic Flexure	MSI
CRC192	Female	36	Adenocarcinoma	Primary CRC	Transverse Colon	MSS
CRC245	Male	64	Mucinous Adenocarcinoma	Liver Metastasis	Splenic Flexure	MSI
CRC403	Male	76	Adenocarcinoma	Primary CRC	Colon	MSS
CRC404	Male	36	Adenocarcinoma	Primary CRC	Rectal	MSS
CRC409	Female	76	Adenocarcinoma	Primary CRC	Colon	MSS
CRC208	Female	68	Adenocarcinoma	Primary CRC	Ascending Colon	MSI
CRCAEH	Anonymous	Anonymous	Adenocarcinoma	Primary CRC		
CRC1001	Male	64	Adenocarcinoma	Liver Metastasis	Sigmoid	MSS
CRC1434	Female	50	Adenocarcinoma	Liver Metastasis	Sigmoid	MSS

Supplemental Table 2 Summary of the mutations in CRC samples

	CRC 106	CRC 187	CRC 192	CRC 245	CRC 403	CRC 404	CRC 409	CRC 208	CRC AEH	CRC 1001	CRC 1434
**ABL1**								*Y257C*	*Y257C*		*Y257C*
**APC**	*R1450** *V1822D* *Q1062fs*		*S1465fs*	*Q1429** *R805**	*R1450**	*R216**	*R1450**		*R1450**	*E1295**	
**CTNNB1**								*S45del*	*S45del*		*A21T* *S45del*
**FLT3**		*T227M* *D7G*									
**KRAS**			*G12D*	*G12D*		*G12D*		*G13D*	*G12D* *G13D*		*G13D*
**PIK3CA**	*H1047L*			*H1047R*			*H1047L*	*H1047R*	*H1047R* *E542K*		*H1047R*
**PTEN**				*R173C* *P96L*							*R173C*
**SMO**								*V404M*	*V404M*		*V404M*
**TP53**	*D228fs* *P72R*	*P72R*	*R175H*	*R181H* *P250H*		*G266E*	*C242Y*			*R342** *P151A*	*R175H*
